# From Nature to Technology: Exploring the Potential of Plant-Based Materials and Modified Plants in Biomimetics, Bionics, and Green Innovations

**DOI:** 10.3390/biomimetics9070390

**Published:** 2024-06-26

**Authors:** Marcela-Elisabeta Barbinta-Patrascu, Bogdan Bita, Irina Negut

**Affiliations:** 1 Department of Electricity, Solid-State Physics and Biophysics, Faculty of Physics, University of Bucharest, 077125 Magurele, Romania; marcela.barbinta@unibuc.ro; 2 National Institute for Lasers, Plasma and Radiation Physics, 077125 Magurele, Romania

**Keywords:** biomimetics, bioinspiration, (nano)bionics, “green” design, phytosynthesis of metal/metal oxide nanoparticles, biomaterials, wastewater treatment, bionic plants, botanical robots

## Abstract

This review explores the extensive applications of plants in areas of biomimetics and bioinspiration, highlighting their role in developing sustainable solutions across various fields such as medicine, materials science, and environmental technology. Plants not only serve essential ecological functions but also provide a rich source of inspiration for innovations in green nanotechnology, biomedicine, and architecture. In the past decade, the focus has shifted towards utilizing plant-based and vegetal waste materials in creating eco-friendly and cost-effective materials with remarkable properties. These materials are employed in making advancements in drug delivery, environmental remediation, and the production of renewable energy. Specifically, the review discusses the use of (nano)bionic plants capable of detecting explosives and environmental contaminants, underscoring their potential in improving quality of life and even in lifesaving applications. The work also refers to the architectural inspirations drawn from the plant world to develop novel design concepts that are both functional and aesthetic. It elaborates on how engineered plants and vegetal waste have been transformed into value-added materials through innovative applications, especially highlighting their roles in wastewater treatment and as electronic components. Moreover, the integration of plants in the synthesis of biocompatible materials for medical applications such as tissue engineering scaffolds and artificial muscles demonstrates their versatility and capacity to replace more traditional synthetic materials, aligning with global sustainability goals. This paper provides a comprehensive overview of the current and potential uses of living plants in technological advancements, advocating for a deeper exploration of vegetal materials to address pressing environmental and technological challenges.

## 1. Introduction

Many living plants are so delicate and seemingly fragile but so resistant to cold, wind, rain, etc. The remarkable ability of plants to adapt to different stress conditions (climatic, drought, flooding, increased salinity, temperature stress, etc.) should be mentioned. Plants developed sensing mechanisms for abiotic stress followed by the downstream cellular and physiological responses [1]. However, plants are immobile living systems, and this fact imposes some limitations. To meet these limitations, plants developed memory and communication abilities, being capable of “adaptively variable behavior”, a term that was proposed by David Stenhouse and then used by Anthony Trewavas [2,3]. The plants are capable of remembering, time measurement, and communication by chemical signals.

Plants play a very important role in the lives of people but also of the majority of terrestrial or aquatic creatures, serving as food, shelter, or as an ornament. Maybe we have passed by certain plants many times, considering them trivial and ignoring them. Countless times we have ignored the leaves, flowers, fruits, or fruit peels that have fallen on the ground and considered them unimportant. Many of these have been thrown in the trash or burned, not knowing that they are actually a valuable source of bioactive substances.

But here, in the last decade, scientists began to use plants and vegetable waste more and more and gave them value, in order to develop ecological strategies to obtain new materials with unexpected properties. Plants are true bionanofactories because they contain many active principles that have a great therapeutic potential, can serve as bioreducing agents for the manufacturing of new nanomaterials, or can be photoactive molecules. Thus, plants have a very wide range of applications in various fields, such as biomedicine, materials science, green nanotechnology, bioelectronics, green electronics, etc.

Moreover, plants served as a source of inspiration for architectural design, in the biomedical field to create artificial organs, or in materials science, nanotechnology, or electronics for developing impressive materials, botanical robotics, biosensors, and so on.

The utilization of plants and vegetal wastes has become increasingly important in recent years due to the pressing need for sustainable solutions to global challenges such as resource depletion, environmental degradation, and climate change.

Plants, with their remarkable diversity and adaptability, play a fundamental role in sustaining life on Earth. Beyond their vital ecological functions, plants offer various applications across numerous fields, ranging from food and agriculture to biomedicine and architecture [4].

This introduction explores the main fields of plant applications, highlighting the necessity of valorizing plants and vegetal wastes to harness their full potential for the benefit of humanity and the planet.

Plants serve as the primary food source for humans and animals, providing essential nutrients, vitamins, and minerals. Agriculture, the cultivation of plants for food, fibers, and other products, relies heavily on plant diversity to meet the growing demand for food in a rapidly expanding global population. Furthermore, plants contribute to producing biofuels, pharmaceuticals, and industrial materials, underscoring their multifaceted role in sustaining agricultural systems and supporting livelihoods worldwide [5]. More than that, weeds (e.g., *Andropogon halepensis*) were used to eradicate other weeds—weeds against weeds—so, as ecological herbicides [6].

In the emerging field of green nanotechnology, plants offer a sustainable alternative to conventional synthetic materials to produce nanomaterials. By leveraging the unique properties of plant-derived nanoparticles (NPs), researchers are developing innovative solutions for environmental remediation, drug delivery, and renewable energy technologies. The eco-friendly nature of plant-based nanomaterials presents exciting opportunities to address pressing environmental and health challenges while minimizing adverse impacts on ecosystems [7].

Plants have long been valued for their medicinal properties, serving as a rich source of bioactive compounds with therapeutic potential. In biomedicine, plant-derived drugs and herbal remedies treat various ailments, ranging from common colds to life-threatening diseases such as cancer and cardiovascular disorders. Moreover, plants hold promise in biomedical engineering, where they are being explored to develop artificial muscles, tissue engineering scaffolds, and biocompatible materials for medical implants [8].

Plants offer a renewable and sustainable source of biomaterials with diverse applications in industry, healthcare, and environmental conservation. From cellulose-based polymers for packaging and textiles to lignin-derived composites for construction and automotive industries, plant-derived biomaterials exhibit superior properties such as biodegradability, biocompatibility, and low environmental impact. By harnessing plants’ inherent properties, researchers are creating innovative biomaterials that address the growing demand for eco-friendly alternatives to traditional materials derived from fossil fuels [9]. Starch, a natural sustainable biopolymer derived from plants, was used as a primary green packaging material as films or coatings on food [10] and also for the preparation of bioplastic for various purposes [11].

The beauty industry relies heavily on plant-based ingredients to formulate skincare, haircare, and cosmetic products. Plants contain many bioactive compounds, antioxidants, and vitamins that nourish and rejuvenate the skin, hair, and nails. From botanical extracts and essential oils to herbal infusions and plant-derived pigments, natural plant ingredients offer safer and more sustainable alternatives to synthetic chemicals commonly found in conventional cosmetics. Moreover, the green and clean beauty trend has fueled the demand for plant-based cosmetics, driving innovation and market growth in this sector [12]. Moreover, plant-based repellents are frequently used today. Thus, the essential oils of basil, peppermint (*Mentha piperita*), turmeric, orange, eucalyptus, cloves, and so on are effective against different Anopheles species [13]. Boonyuan et al., reported that citronella (*Cymbopogon nardus* L.) oil showed promising repellent activity against several female mosquitoes [14].

Advancements in genetic engineering have transformed plant breeding, allowing for the development of disease-resistant, drought-tolerant, and nutritionally enhanced crops. Researchers use gene editing to introduce beneficial genes, improving agronomic performance and nutritional value. Plants are also engineered to produce high-value bioproducts, such as silk proteins, for industrial and biomedical uses [15].

Plants offer a sustainable and biocompatible platform for developing green electronics, where organic materials derived from plants are used to fabricate electronic devices such as sensors, transistors, and energy storage systems. By harnessing the unique properties of plant-derived materials such as cellulose, lignin, and chlorophyll, researchers are creating biodegradable and environmentally friendly alternatives to conventional electronic materials based on non-renewable resources. Green electronics hold promise for reducing electronic waste and minimizing the environmental footprint of electronic devices, contributing to a more sustainable and circular economy [16].

Plants possess inherent biological mechanisms that enable them to detect and respond to environmental changes, making them ideal candidates for biosensor applications. Plant-based biosensors utilize living plants or plant-derived materials to detect specific analytes or environmental pollutants in real-time, offering cost-effective and noninvasive solutions for environmental monitoring, food safety, and healthcare diagnostics. By interfacing plants with nanomaterials or electronic sensors, researchers can enhance the sensitivity and selectivity of plant-based biosensors, opening up new possibilities for rapid and portable detection technologies [17].

Plants have been used for millennia to produce natural fibers for textiles, clothing, and home furnishings. From cotton and linen to hemp and bamboo, plant-based fibers offer superior comfort, breathability, and sustainability compared to synthetic fibers derived from petrochemicals. Moreover, plants are a rich source of natural dyes and pigments, providing a renewable and eco-friendly alternative to artificial dyes that often contain harmful chemicals and pollutants. By embracing plant-based textiles, consumers can reduce their environmental footprint and support ethical and sustainable practices in the fashion and textile industry [18]. Furthermore, bionic textiles have been developed through bio-inspiration from superhydrophobic plants [19].

Today, there are many phyto-based approaches such as using plant-derived biomolecules, entire living plants, and the phytosynthesized nanomaterials for wastewater treatment. One of these methods is phytoremediation, i.e., the use of living plants in wastewater treatment. Phytoremediation is an emerging technology that uses photoautotrophic vascular plants for the clean-up of sites (soil and groundwater) contaminated with inorganic and organic contaminants [20]. Another approach is using biomolecules derived from vegetal waste for the degradation of water pollutants. For example, unpurified peroxidase extracted from cabbage leaf was used for phenol removal from polluted water [21]. The third method consists of using the plant-derived nanomaterials such as ZnO NPs and CuO NPs for wastewater treatment [21].

Plants have been integral to architectural and home design since the ancient times, providing building materials, insulation, and aesthetic elements for structures ranging from traditional dwellings to modern skyscrapers. Wood, derived from trees, is one of the most versatile and sustainable building materials.

In the following sections, some of the unusual applications of plants will be detailed.

## 2. Plants in Materials Science

### 2.1. Plants as Inspiration in Developing Materials

Plants have long served as a rich source of inspiration for innovative materials and technologies, exemplified by several fascinating adaptations emulated in human-made products.

Plants are immobile organisms, remaining at the location where they sprout from seeds throughout their lifespan. They have developed a great degree of plasticity, allowing them to flourish, adjust, and react to changing surroundings, enabling survival under stress [22]. With their remarkable adaptability, plants are the initial organisms to inhabit challenging conditions. They possess the special ability to thrive in two distinct environments simultaneously, such as soil and air or water and air [23]. Organisms’ behaviors are shaped by their physical structure, sensory-motor control, and environmental interactions, known as “morphological computation”. This concept emphasizes the importance of physical properties and organization in behavior. Plants serve as excellent models for robotic operations in unstructured environments due to their advanced observation skills, efficient energy use, and adaptability. They offer insights for developing advanced technologies, including continuous growth, motion without muscles, adaptable materials, decentralized intelligence, effective anchoring, robust communication, and energy-efficient mechanisms. Plant roots explore soil non-destructively, efficiently seeking nutrients, while aboveground, they inspire the design of lightweight, compact robots that can anchor, navigate tight spaces, and climb where traditional robots might fail. These abilities make plants ideal models for innovative robotic designs, providing solutions for navigation and stability in challenging conditions.

**Figure 1:**
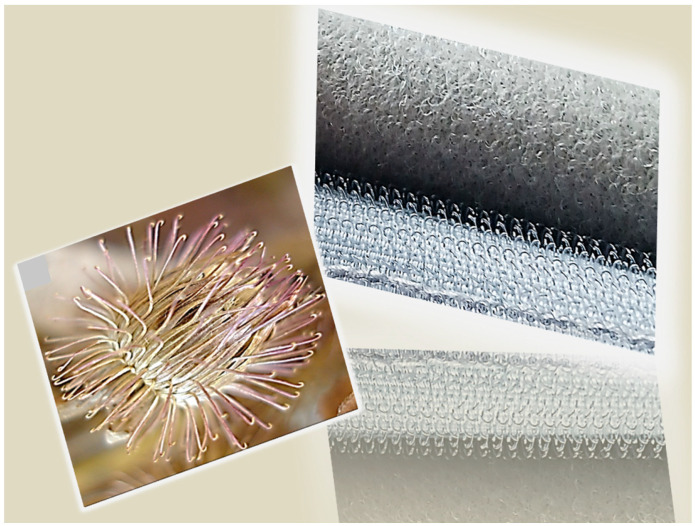
Burrs of the burdock (*Arctium lappa*) plant (left) and Velcro (right).

One of the most widely recognized plant-inspired inventions is Velcro (the hook-and-loop fastener). The concept for this now ubiquitous fastening mechanism came from the burrs of the burdock (*Arctium lappa*) plant (Figure 1), which routinely attached themselves to the clothing and fur of passersby [24]. In 1941, during a hunting trip in the Swiss Alps, the Swiss engineer George de Mestral noticed how burdock burrs stuck to his pants and to his dog’s fur. George de Mestral, intrigued by the tenacity of these burrs, examined them under a microscope and discovered their secret: tiny hooks that easily latched onto the loops of fabric or fur. This observation led to the creation of Velcro, a hook-and-loop fastener system that mimics the burr’s natural attachment mechanism [25]. The etymology of the word Velcro originates from the French words “Velours” (velvet) and “Crochet” (hook) and it became popularized after NASA used the technology for the space costumes of their astronauts [25].

**Figure 2:**
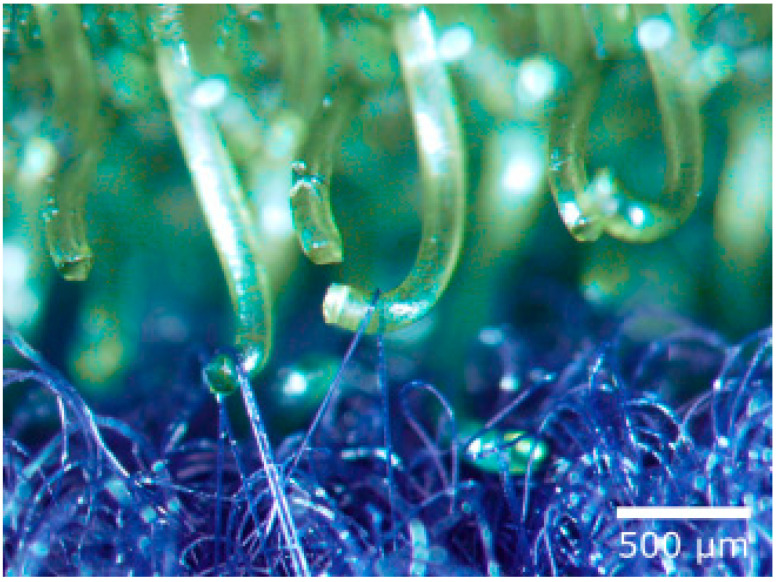
Hook-and-loop fastener (Velcro^®^) with hook tape (top) and loop tape (bottom) [24].

Velcro consists of a hook tape and a loop tape (see Figure 1, right). The hook tape has a large number of elastic hooks and it resembles the burrs of the burdock. The loop tape looks like animal fur and consists of a large number of fine closed loops which can become trapped in the hooks (Figure 2) [24].

**Figure 3:**
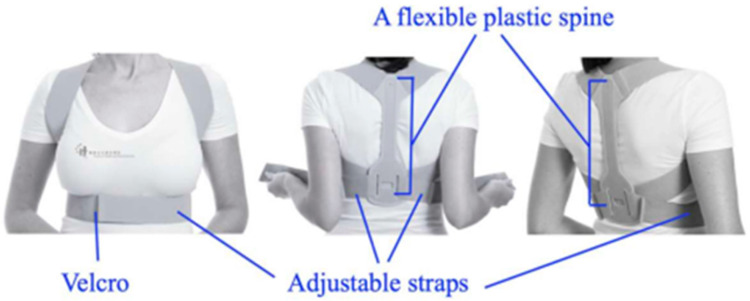
A commercially available scapular brace used to improve upper back/neck posture [26].

Velcro is one of the best-known and commercially successful biomimetic materials which is used in various fields such as the biomedical, textiles, footwear, prosthesis fields, and so on. Thus, Velcro straps have been employed in postural braces used to restore normal scapular posture and scapular muscle activation (Figure 3) [26].

**Figure 4:**
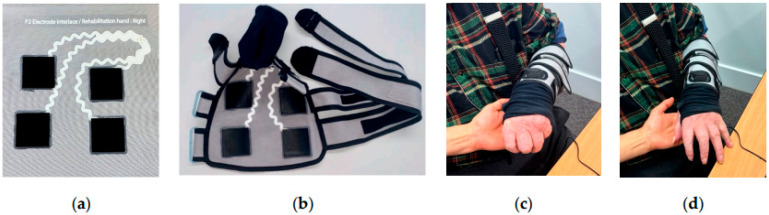
(**a**) Sine wave track with pads and wearable carbon electrode on all needle back fabrics; (**b**) an e-sleeve with an open and Velcro closure structure; (**c**) a stroke hand in the relaxation mode; (**d**) the stroke hand achieved an open gesture with FES stimulation [27].

Velcro is also used for attaching wearable systems such as e-sleeves with an open and Velcro closure structure (Figure 4) for stroke rehabilitation by functional electrical stimulation (FES) [26].

**Figure 5:**
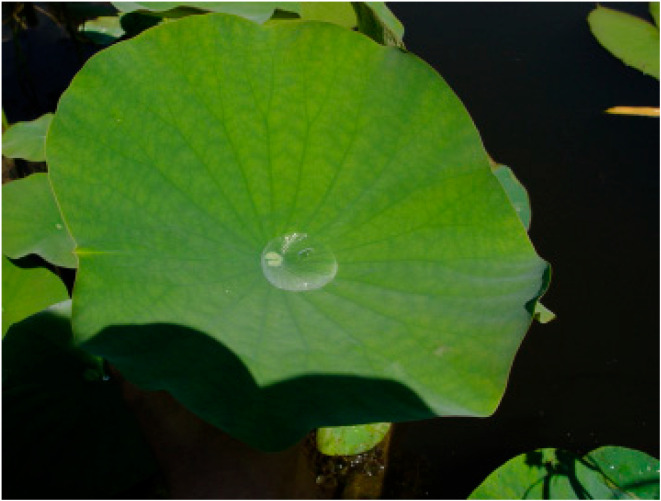
A spherical drop of water formed on the hydrophobic surface of the Indian lotus (*Nelumbo nucifera*) leaf [24].

**Figure 6:**
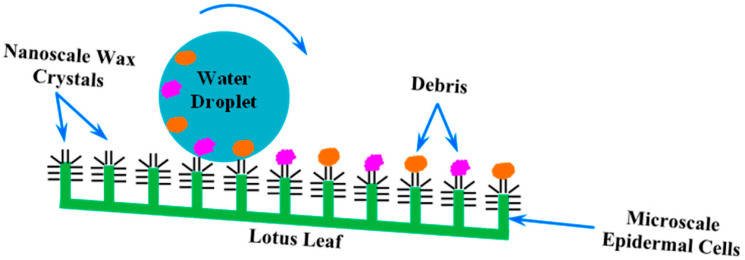
An easy-to-understand schematic of the Lotus Effect: As the water droplet rolls off the leaf, it gathers debris along its path [29].

Another remarkable example is the lotus flower, renowned for its incredible self-cleaning properties. The surface of the lotus leaf is extraordinarily hydrophobic, causing water droplets (Figure 5) to bead up and roll off, picking up dirt and contaminants along the way. This phenomenon, known as the “Lotus Effect” (Figure 6), has inspired a range of water-repellent surfaces. The “Lotus Effect” therefore refers to the self-cleaning property of lotus leaves. The leaf surface is covered with microscopic bumps coated with a hydrophobic waxy substance, causing water droplets to bead up and roll off, taking dirt particles with them. This phenomenon inspired the development of self-cleaning materials and surfaces, including paints and coatings used in buildings and textiles. These materials mimic the lotus leaf’s microstructure to achieve water repellency and self-cleaning properties [28]. Materials engineered to replicate the micro- and nanostructures of the lotus leaf surface are now used in a variety of applications, from self-cleaning windows and paints to waterproof fabrics and anti-stain materials [24,29,30].

Moreover, the superhydrophobic leaves of *Salvinia molesta* adsorb oil and separate it from water surfaces. This phenomenon inspired the scientists who created textiles with a bionic oil adsorber [19].

**Figure 7:**
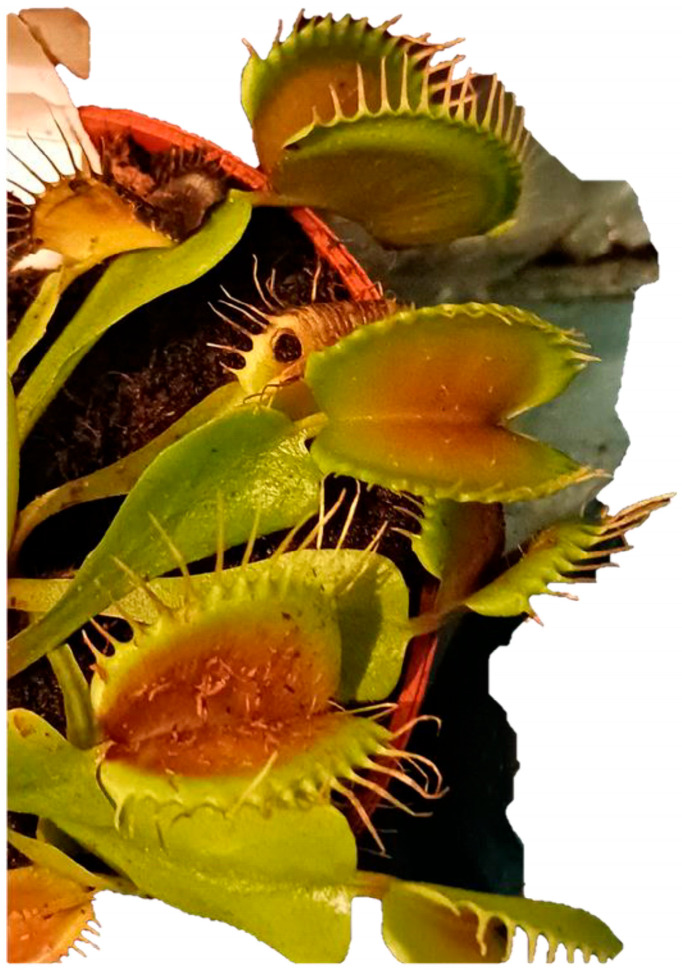
The picture of the carnivorous plant *Venus flytrap* (*Dionaea muscipula*).

The *Venus flytrap* (Figure 7), a carnivorous plant, has also provided biomimetic inspiration with its rapid leaf movements that trap prey. The *Venus flytrap* captures prey by rapidly closing its leaves when trigger hairs are touched. This fast movement results from the rapid change in turgor pressure within the leaf cells. Engineers have mimicked this mechanism to develop soft robotic grippers capable of delicate and precise movements. These robots are made from flexible materials and use changes in air pressure or hydraulic systems to mimic the closing motion of the *Venus flytrap*, allowing them to handle fragile objects without damage. The mechanisms by which these plants detect and ensnare their prey have led to advancements in responsive materials and structures in robotics and engineering. Researchers are studying the biomechanics of the *Venus flytrap* to develop new types of sensors, actuators, and more efficient robotic mechanisms that can respond dynamically to changes in their environment [31,32].

**Figure 8:**
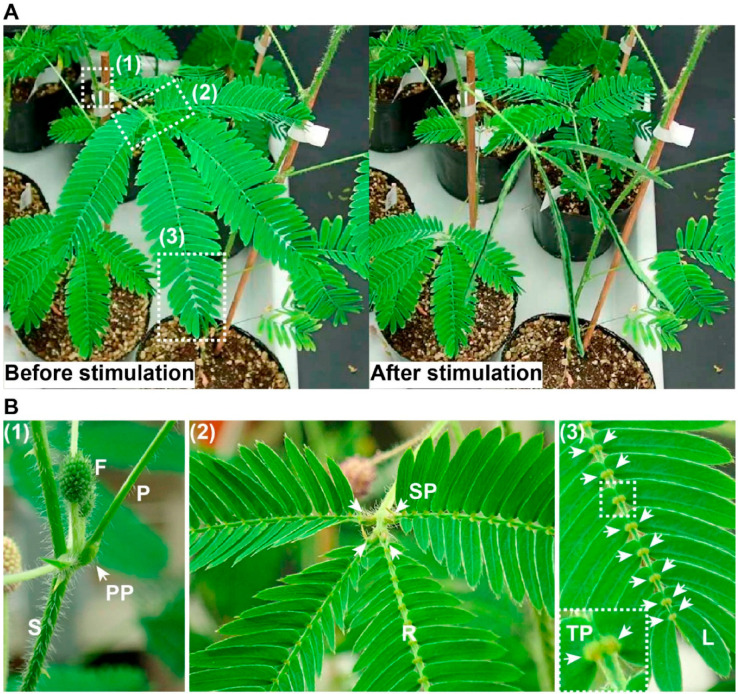
Characteristics of rapid movement in *Mimosa pudica* are illustrated in the following manner: (**A**) The leaf of *Mimosa pudica* is shown fully expanded before mechanical stimulation and folds upon touch. (**B**) The motor organs, or pulvini, are detailed: (1) primary pulvinus (PP, white arrow), with components labeled as stem (S), floral bud (F), and petiole (P); (2) secondary pulvini (SP, white arrows), associated with the central axis of the pinna (rachilla, R); (3) tertiary pulvini (TP, white arrows), with an inset magnifying pairs of tertiary pulvini enclosed by a dashed line. The numbers in each panel correspond to those in (**A**) [34].

Certain plant species exhibit rapid movement in response to external stimuli. For instance, the sensitive leguminous plant *Mimosa pudica* swiftly folds its leaves upon mechanical stimulation. Extensive research has documented the propagation of stimulus information induced by such mechanical stimuli. This rapid movement and long-distance signaling have been central to significant research endeavors, though considerable gaps remain in our understanding of the underlying mechanisms. This review aims to explore the current consensus on mechanical signaling responsible for rapid movement and long-distance signal propagation in *Mimosa pudica*. When a leaf of *Mimosa pudica* is mechanically stimulated, such as by touch, it drops and the leaflets fold upward, an action facilitated by pulvini located at the base of the leaflets, rachilla, and petiole. Among the three types of pulvini, the primary and tertiary are more sensitive to mechanical stimuli than the seconddary. Early research showed that movement still occurred even when the flexor side of the primary pulvinus was removed, suggesting the extensor side’s crucial role. Further studies confirmed that both extensor and flexor sides are essential for movement. This is driven by the redistribution of water from the extensor to the flexor side, causing sudden turgor loss in the extensor side, which powers the rapid movement (Figure 8) [33].

Novel research introduces the Mimosa kinetic façade, an innovative design inspired by the Mimosa plant’s adaptive response to environmental stimuli. Unlike traditional static façades that impede natural ventilation and degrade air quality, this dynamic façade improves airflow and removes airborne contaminants. Utilizing the biomimicry design spiral, the research adopts a nature-inspired approach to enhance both the functional and visual aspects of building design [35].

The design of seeds that can float on the wind for miles, like those of the dandelion, has inspired the development of lightweight, aerodynamically efficient structures in aerospace engineering.

Dandelion seeds have a unique structure featuring a parachute-like bundle of bristles called a pappus, which increases air resistance and enables the seed to be carried by the wind over long distances. This efficient wind dispersal mechanism has inspired the design of passive flying devices, such as lightweight drones or sensors that can be dispersed over large areas by the wind for environmental monitoring. Researchers studied the aerodynamics of the dandelion seed to optimize the design of these devices for stability and prolonged flight [36]. Dandelion seeds achieve remarkable stability and distance by optimizing their shape and surface area, which minimizes air resistance and enhances lift. Engineers have studied these properties to create advanced materials and structures that mimic these aerodynamic principles, leading to innovations in drone design, parachutes, and even lightweight spacecraft components. This biomimetic approach exemplifies how natural solutions can address complex engineering challenges, promoting efficiency and sustainability [37]. For instance, researchers at the University of Washington developed tiny, battery-free wireless sensors that mimic the dandelion seed’s ability to travel great distances by wind. These sensors are designed to be lightweight and aerodynamically efficient, allowing them to disperse over large areas when released by drones. This innovation is particularly useful for environmental monitoring and agricultural applications, where sensors need to be deployed over vast, hard-to-reach areas. The structure of dandelion seeds, which includes a central point with radiating bristles, has been key to these developments. This design slows their descent, increasing the distance they can travel and ensuring they land in an optimal orientation. Engineers have adapted these principles to create sensors that can monitor environmental conditions such as temperature, humidity, and light. These sensors use solar panels for power and can transmit data wirelessly, leveraging a method called backscatter to send information to researchers [38].

Each of these examples demonstrates how plants have evolved sophisticated strategies to thrive in their environments, providing valuable lessons for developing materials that are not only functional but also sustainable and efficient. As we advance our capabilities in biomimicry and biologically inspired engineering, the potential to harness and expand upon these natural designs holds promising solutions to many of today’s engineering and environmental challenges.

### 2.2. Phyto-Nanotechnology

Biocomposite NPs incorporating plant extracts represent a fascinating intersection of nanotechnology and natural product chemistry. These NPs are synthesized by integrating plant-derived compounds into a bio-matrix (such as biomimetic cell membranes, spider silk, etc.), often enhancing the properties of the NPs [39,40,41].

**Figure 9:**
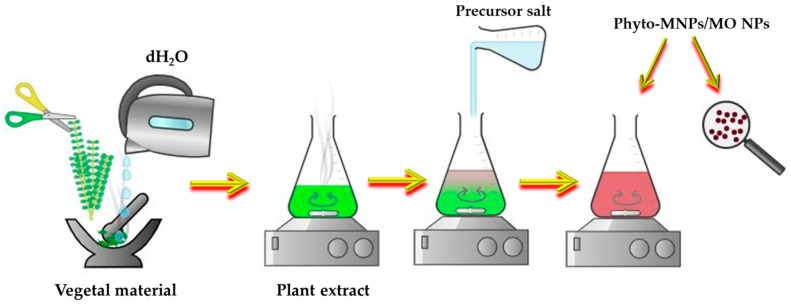
Schematic representation of the phytosynthesis of metal nanoparticles and metal oxide nanoparticles. The figure was created with Chemix (https://chemix.org/, accessed on 25 February 2024) and with PowerPoint and Paint 3D.

The plant materials are often used for the biosynthesis of NPs, since the plants and their wastes are eco-friendly, easily available, low-cost, and can be reused. This approach, called *phytosynthesis*, is an easily upscalable, bottom-up strategy which involves the use of a “green” solvent—water—to obtain the phyto-extract and a metal salt precursor for the phyto-development of NPs (Figure 9). This method respects the 12 Principles of “Green Chemistry”, elaborated by Anastas and Warner in 1998 [42].

The creation of biocomposite NPs incorporating plant extracts involves several key processes, starting with the preparation of the plant extract. Initially, plant materials such as leaves, stems, roots, or flowers are collected and thoroughly washed. Once cleaned, the plant material is dried and ground into a fine powder. This powder undergoes extraction of bioactive compounds using solvents like water, ethanol, or methanol through methods such as maceration, Soxhlet extraction, or ultrasound-assisted extraction. Following the extraction, the NP synthesis process begins. The plant extract is combined with a precursor solution containing metal ions, such as silver nitrate for silver NPs. Reduction agents within the plant extract, including polyphenols, flavonoids, and alkaloids, facilitate the reduction of metal ions, leading to the formation of NPs. This synthesis can occur at room temperature or under controlled heating and stirring conditions to ensure that the NPs have a uniform size and shape [43].

The biomolecules arising from vegetal extracts (e.g., polyphenolic compounds, proteins, saponins, polysaccharides, tannins, alkaloids, phytopigments, etc.) act as an electron shuttle for reducing the metal ions. They can serve both as reducing agent and also as capping and stabilizing agents in the phytosynthesis of MNPs/MO NPs [44]. Moreover, the phyto-molecules coating the surface of phytogenic NPs impart interesting properties of developed MNPs/MO NPs such as biocompatibility, antimicrobial activity, antioxidant properties, and anticarcinogenic properties. These biological components mentioned above form a surrounding layer around the individual NPs called the “capping layer” or “biological corona” which is responsible for the bioactivities of NPs and for the long-term stability of NPs in aqueous media, protecting the NPs from agglomeration, and playing an important role in the interaction of “green” NPs with living cells [45]. Therefore, these NPs can be used in various biomedical applications such as cancer theranostics, tissue engineering, drug/gene delivery, wound healing, and antimicrobial use. Moreover, the phytosynthesized MNPs/MO NPs have been also applied in cosmetics, nanosensors detecting biomolecules, nanobiopesticides, and photocatalysts [44,46].

For the plant-based synthesis of NPs, leaves are preferred due to their special composition rich in photopigments (e.g., chlorophylls and anthocyanins) and other molecules (terpenes and flavonoids) with strong bioreducing action. However, other plant parts are used such as flowers, roots, seeds, fruit, callus, bark, and stem. Compared with microbial-based synthesis, MNPs/MO NPs obtained using phyto-extracts have higher stability and a faster reaction time.

The most popular MNPs/MO NPs are AgNPs, AuNPs, CuO NPs, and ZnO NPs. These NPs exhibit interesting properties alone and in composites as well, and they have been used in various applications (biomedical, agricultural, nanotechnological, materials science, etc.). Additionally, their biocomposites possess novel properties and functionalities that open up new possibilities for innovative applications in different fields [39,47].

In agriculture, the phytosynthesized NPs can also be used as nanofertilizers and nanopesticides [45,46]. MNPs/MO NPs have been explored as nanobionics in crop production to enhance the photosynthesis rate [48].

Indeed, the benefits of using MNPs/MO NPs depend on dose and size. When overcoming a dose limit, they can induce harmful effects.

Among the many plants used for the green synthesis of MNPs and MO NPs, *Mentha* species have more attractiveness. This is due to the fact that these aromatic and medicinal plants contain many bioactives such as rosmarinic acid, hesperidin, and eriocitrin with interesting properties (e.g., antioxidant, anti-inflammatory, antimicrobial, and antiallergic properties) [49].

AgNPs biosynthesized using *Mentha piperita* (mint or peppermint) extract were embedded into a cellulose membrane at a low concentration. The obtained cellulose-AgNP membrane proved to be an excellent substrate for the remediation of pesticide-polluted water [50].

Packialakshmi et al., described a green synthesis of ZnO NPs using a *Mentha piperita* leaf extract. The obtained ZnO NPs were employed as a phytocatalyst to make chitosan derivatives [51]. Another research group phytosynthesized ZnO NPs using an aqueous extract of aerial parts of *Mentha pulegium* L. The phyto-developed ZnO NPs were used to develop composites with copolymer 2-Hydroxyethyl methacrylate (HEMA)-co-2-oxo-2-(3,4,5-trifluoroanilino)-ethyl-2-methylprop-2-enoate (FAOEME) (Poly(HEMA-co-FAOEME)) [52]. These nanocomposites showed antimicrobial activity and wound healing properties due to the green ZnO NPs’ presence.

Stoyanova et al., reported the green synthesis of nanosized ZnO particles using an extract of *Mentha arvensis* leaves as a stabilizing/reducing agent, followed by hydrothermal treatment at 180 °C [53]. The obtained ZnO NPs presented photocatalytic activity for the degradation of Malachite Green dye.

Green CuO NPs fabricated using mint leaves proved to be an adsorptive nanomaterial to purify polluted water from heavy metals such as Pb(II), Cd(II), and Ni(II) [54].

CuO NPs synthesized using mint leaf extract were more bioperformant than green CuO NPs synthesized using *Eucalyptus* leaf extract against the fungus *Colletotrichum capsica* [55].

### 2.3. Camouflage Inspiration from Green Vegetation

Vegetation serves as the predominant environmental backdrop, exhibiting distinctive spectral characteristics that enable its differentiation from other elements like rocks, water, sand, and anthropogenic objects. These unique spectral features of vegetation are pivotal for various applications, including remote sensing and ecological monitoring, facilitating the identification and analysis of vegetative cover in contrast to inanimate and man-made surfaces. Camouflage patterns, designed to mimic the appearance of natural backgrounds, hold significant practical value in both civilian and military contexts, aiding in concealing targets from traditional optical/visual observation [56,57,58]. However, the advent of advanced detection technologies, particularly hyperspectral imaging, challenges the effectiveness of conventional camouflage by detecting spectral discrepancies between the target and its surroundings [59]. This technological evolution necessitates the development of camouflage materials that not only match the visual appearance of their background but also closely replicate its spectral characteristics across the visible to near-infrared (Vis–NIR) spectrum (380–2500 nm), raising the bar for camouflage effectiveness against modern detection methods [60].

The green hue of natural leaves primarily arises from the interplay between chlorophyll’s light absorption *in vivo* and the light’s internal reflection at the membrane interface [61]. While modern colorants can mimic the chromaticity values of leaves to replicate their appearance under specific lighting conditions, replicating the exact reflectance spectrum of green leaves poses a significant challenge [61]. This difficulty stems from the unique coloration mechanism of leaves, which often leads to artificial greens being distinguishable from their natural counterparts under varied lighting conditions or hyperspectral imaging [59]. Despite the diversity of leaf species, the green coloration and spectral characteristics are generally consistent across species, attributed to the chloroplast, a key photosynthetic organelle [62]. Chloroplasts feature a microcapsule structure containing grana consisting of lipids, proteins, and chlorophyll pigments [63]. The arrangement of pigment–protein complexes and the “package” effect result in chloroplast’s *in vivo* pigment absorption spectrum differing markedly from that of extracted pigments [64].

The selective light attenuation by *in vivo* pigments and reflection by internal cell walls create a distinctive foliar reflectance profile. Notably, the dominant green coloration of leaves is due to the reflectance peak around 550 nm [65], influenced by the absorption of chlorophyll and carotenoids *in vivo* [65]. Different types of plant leaves share similar spectral reflectance characteristics despite their variety. A notable feature within the visible spectrum is the “green peak”, primarily due to chlorophyll pigments’ minimal absorption of green light, rendering the leaves green [66]. Between 700 and 1300 nm, features such as the “red edge” and “near-infrared plateau” emerge, attributed to the scattering of solar radiation within the multi-layered cellular structure of leaves. The “red edge” is an abrupt shift in reflectance between 680 and 780 nm [67], arising from the combined effects of chlorophyll’s narrow Q-band absorption [65], the compartmentalized environment sustaining the chlorophyll [68], and the internal reflection at cell walls, which enhances reflectance at wavelengths not absorbed by chlorophyll [65]. In addition, the width of the red edge can be used as an indicator to estimate the water content of plants with a certain level of confidence. This measurement reflects the plant’s physiological state and hydration levels, providing valuable insights for environmental and agricultural studies [69]. Further into the spectrum, from 1300 to 2500 nm, “water absorption valleys” are observed, which are caused by the absorption of radiation by water contained in the leaf’s mesophyll tissue [70]. These spectral features are fundamental to understanding plant physiology and the interaction of vegetation with light across different wavelengths.

Therefore, the Vis–NIR camouflage materials can be divided into the following: (i) simulating the NIR and Vis spectrum of plant leaves to obtain camouflage materials with Vis–NIR spectral performance and (ii) color performance. Various bionic materials have been developed to imitate the reflection characteristics of plant leaves in the solar spectrum waveband by realizing the absorption and scattering processes with the components and structure of the materials.

Extracting chlorophyll to color different polymer matrices has been explored to replicate leaf reflectance [63]. However, the spectral and chemical degradation of chlorophyll outside the *in vivo* environment leads to a redshift of the red edge, compromising the spectral similarity to natural leaves and limiting the potential for patterning and scaling these matrices [63]. Commercial green pigments, specifically Cr_2_O_3_ (C.I. Pigment Green 17) and CoCr_2_O_4_ (C.I. Pigment Green 26), offer excellent manufacturability and durability but suffer from spectral discrepancies compared to foliage. Cr_2_O_3_, despite having a peak reflectance centered around 540 nm that can be adjusted by doping, exhibits a milder slope in the red edge region, diminishing its likeness to leaf spectra [71,72]. While closely matching the slope of the red edge, CoCr_2_O_4_ presents undesirable characteristics due to its blue-shade color and strong absorption in the NIR region attributed to tetrahedrally coordinated cobalt. Additionally, commercial disperse dyes have been used to approach foliar reflectance but face challenges due to environmental impacts on the dye molecules. These impacts can include dye–dye aggregation, dye–fabric bonding, and light scattering by fabric fibers, often resulting in low Vis reflectance, dark brownish coloration, and mild slopes in the red edge region.

To overcome these challenges, an “ideal” spectral simulation strategy would need to incorporate chlorophyll-like absorption characteristics, maintain these characteristics within a suitable environment, and feature a compartmentalized pigment structure amenable to painting and patterning. This approach should also ensure compatibility with various substrates to close the gap between artificial and natural leaf spectral properties.

Drawing inspiration from the natural reflectance characteristics of plant leaves, a recent study introduces a bionic coating composed of chromium oxide (Cr_2_O_3_) particles and a hydrophilic inorganic salt embedded within a polyvinyl alcohol (PVA) matrix, applied to metal plates [73]. The Cr_2_O_3_ particles mimic the absorption properties of chlorophyll and the scattering effects observed in plant leaves’ multilayer cellular structure, enabling the coating to replicate leaf reflection characteristics across the visible and near-infrared spectrum. Additionally, including hydrophilic inorganic salt allows the coating to absorb atmospheric moisture, simulating the water absorption valleys characteristic of plant leaves by absorbing incident radiation. The optimal suppression of metal plate reflectance in the solar spectrum was achieved with a Cr_2_O_3_ particle volume fraction of 0.81% and a coating thickness exceeding 0.2 mm. Enhancing the metal plates’ surface roughness further improved performance between 800 and 1300 nm, aligning the reflection curves of the bionic coating more closely with those of plant leaves. The coating’s ability to accurately mimic critical plant leaf reflection characteristics underscores its significant potential for applications in hyperspectral camouflage [74]. In a similar matter, hydrophilic camouflage coating incorporating Cr_2_O_3_ green pigment particles was applied to a stainless-steel substrate, aiming to replicate the reflectance characteristics of plant leaves [74]. The pure PVA coating’s reflectance in the 1300 nm to 2500 nm range closely resembled that of leaves. By integrating Cr_2_O_3_ particles, the coating could simulate leaves’ four fundamental reflectance traits. As the volume fraction of Cr_2_O_3_ increased, the underlying optical properties of the stainless-steel substrate had a diminishing effect on the coating’s reflectance. At a Cr_2_O_3_ volume fraction of 0.8%, the coating effectively concealed the stainless-steel substrate’s reflectance characteristics, achieving camouflage.

Moreover, the coating’s reflectance improved with moisture absorption from humid environments due to two primary effects: a reduction in the refractive index, enhancing reflectance between 800 nm and 1300 nm, and a significant increase in the absorption coefficient around 1460 nm and 1940 nm. These changes reduced the coating’s reflectance in these regions, aligning more closely with leaf reflectance. The concealment of the stainless-steel substrate’s reflectance was optimized by increasing the Cr_2_O_3_ content. At the same time, adding LiCl enhanced water absorption bands’ visibility, further mimicking leaves’ spectral characteristics [74].

Hu et al., developed a bionic polyurethane material (PU-SSM) engineered to replicate the spectral characteristics of plant leaves. This innovative material, designed with a specific void ratio and density, emulates the structure of leaf flesh. Incorporating two types of colorants—paint and reduction dyes—mimics the chlorophyll found in leaves, while the material’s water content simulates the moisture inherent in natural leaves. This bionic approach enables the material to exhibit spectral characteristics remarkably similar to those of tree leaves, including the green peak, red edge, near-infrared plateau, and water absorption valleys, achieving spectral correlation coefficients as high as 0.984. Furthermore, this study contributes to the broader field of infrared camouflage bionics by focusing on the functional aspects of leaf mimicry [75].

Inspired by the natural process of leaf chromogenesis, Xie et al., devised and verified an innovative artificial spectral simulation colorant and methodology, showcasing the unparalleled potential for practical camouflage applications [76]. The team fabricated a microcapsule colorant to display the absorption of monomeric zinc phthalocyanine (ZnPc). The monomeric ZnPc provides highly selective absorption, like the *in vivo* chlorophyll Q-band. ZnPc was stabilized within an aprotic polar environment, mirroring the natural environment chlorophyll enjoys within the grana of plant cells. This stabilization approach is analogous to how cell walls compartmentalize plant cells, safeguarding cellular components from external influences [77]. Encapsulating monomeric ZnPc within a microscale polymer shell effectively isolates the internal environment from external disturbances. The transparent polymer shell aids internal reflection, mimicking natural leaf reflectance. This design precisely tunes the red edge of the microcapsules to match natural leaves, advancing biomimetic materials for spectral simulation. These microcapsules ensure durable camouflage under high temperature and humidity in forests. The bilayer structure allows for independent adjustment of the red edge and NIR reflectance, accurately replicating natural foliage’s spectral diversity. This adaptability enhances camouflage across the Vis, NIR, and IR spectra, providing robust defense against hyperspectral detection and potentially leading to misidentification as natural leaves.

Lu et al., took inspiration from the transpiration process of plant leaves to create a self-driven, water-absorbing bionic leaf. This innovative bionic leaf was fabricated using a combination of hygroscopic calcium chloride (CaCl_2_) and sodium alginate cross-linking. This design enables the bionic leaf to effectively simulate the visible to near-infrared (Vis-NIR) spectral characteristics of natural plant leaves, showcasing the potential of biomimetic approaches in replicating the unique properties of vegetation [78].

Green chlorophyll, while a primary pigment, is not the sole colorant present within leaf cells; other pigments like yellow xanthophylls and orange beta-carotene also play significant roles. In the growth season, chlorophyll predominates due to its crucial function in photosynthesis, effectively concealing the presence of other pigments [79]. However, as daylight hours diminish and temperatures drop, photosynthetic activity decreases, reducing chlorophyll levels [79]. Eventually, chlorophyll breaks down into colorless tetrapyrroles, unveiling the previously masked pigments of xanthophylls and beta-carotene, contributing to the color changes observed in foliage during the autumn season [80].

Based on the leaf color change process, Huang et al., proposed a mimicking color-change model through the development of a color-changing biomimetic material [81]. This material integrates active micro-temperature-responsive pigments (MTPs) and static color-tunable yellow pigments (CTYPs) within a polyvinyl alcohol (PVA) and lithium chloride (LiCl) matrix. Utilizing the Kubelka–Munk four-flux model and a genetic algorithm, the color-changing biomimetic material (CCBM) was crafted *via* solution casting to replicate the UV–vis–NIR spectra and thermal infrared characteristics of both green and yellow leaves. The CCBM displayed distinctive spectral features identical to natural leaves, including the “green peak”, “red edge”, and “near-infrared plateau” in its green state and “green edge”, “near-infrared plateau”, and “water absorption valley” in its yellow state. Spectral correlation coefficients between the CCBM and various plant leaves, including Gardenia, Osmanthus, Magnolia, Money plant, and Ginkgo, ranged from 0.96 to 0.99, indicating high similarity. This resemblance extended to the grayscale values in multispectral imaging, closely matching the colors of Ginkgo leaves in both green and yellow states. Outdoor testing revealed that the thermal infrared characteristics of the CCBM closely aligned with those of natural leaves, with minimal temperature differences observed, showcasing its potential for thermal camouflage. The material’s color could be altered through external temperature stimuli, exhibiting bistable characteristics [81].

In addition to structure-based biomimetic research, studies have delved into infrared camouflage bionics, focusing on replicating the functional aspects of leaves. Xu et al., have innovatively designed bionic materials composed of hydrophilic PVA and lithium chloride. These materials can emulate the natural transpiration cooling effect observed in leaves, offering a strategic countermeasure against thermal infrared detection. By leveraging moisture desorption, these bionic materials can autonomously regulate their temperature, employing a transient heat mass transfer model to mimic the thermoregulatory functions of leaves effectively [82].

## 3. Plant-Based Sources for Tissue Engineering

Tissue engineering, an interdisciplinary field integrating regenerative medicine, materials science and engineering, and cell biology, has markedly advanced medical practices by enhancing bodily functions and replacing damaged tissues [83]. At its heart, this field focuses on creating 3D cellular and biomaterial complexes to recreate the intricate environment necessary for tissue growth and function. The pivotal aspect of tissue engineering is its capacity to induce cellular responses to morphogens, scaffolds, and various signals, encompassing three main strategies: cell-based, signal-based, and scaffold-based approaches [84]. Scaffolds, in particular, serve as an artificial extracellular matrix to support cell growth towards forming complete tissue structures. In contrast, growth factors are crucial in directing and harmonizing the cellular activities within the developing tissue [83].

As mentioned above, the first tissue engineering strategy represents the creation of biocompatible scaffolds that provide a conducive environment for cell attachment, proliferation, and differentiation, ultimately supporting tissue regeneration [85]. These scaffolds offer mechanical stability, elasticity, and strength and, importantly, mimic the microenvironment necessary for the growth of seed cells [86]. Given that the biology of the surrounding microenvironment critically influences the implanted cells’ behavior, scaffold properties are of utmost importance. Consequently, extensive research has been dedicated to exploring the composition, structure, function, and fabrication techniques of scaffolds [87].

Ideal tissue engineering scaffolds should be biocompatible, biodegradable, possess high porosity and interconnectivity, and maintain mechanical integrity [88]. The structure and shape of scaffolds are also critical, as they can guide the regenerated tissues’ structure, size, and morphology. Scaffolds are broadly categorized into natural and synthetic types [89]. Natural scaffolds are made from polymers like collagen, silk fibroin, and hyaluronic acid. In contrast, synthetic scaffolds are composed of polymers such as polyglycolic acid (PGA) [90], polylactic acid (PLA) [91], polylactic-co-glycolic acid (PLGA) [92], and polycaprolactone (PCL) [93].

Despite these advancements, current scaffold materials face challenges, including insufficient structural biomimetics and a need for cell adhesion sites in synthetic scaffolds [94]. These limitations underscore the need for scaffold design and materials innovation to replicate the natural extracellular matrix (ECM) better and support tissue engineering goals [95].

Tissue engineering scaffolds act as a crucial interface between cells and tissues, effectively mimicking the ECM to guide tissue formation [96]. The ECM, an ideal model for tissue scaffold biomaterials, is vital in maintaining cellular structure and modulating cell growth, proliferation, orientation, and differentiation. It also emits signals critical for regulating cell survival, migration, proliferation, and differentiation, facilitated by various bioactive substances like growth factors and cytokines.

Given the ECM’s complexity, including its diverse components like proteins and glycosaminoglycans, replicating its composition and intricate ultrastructure *in vitro* presents substantial challenges [97]. Conventional physicochemical methods struggle to reconstruct scaffolds that accurately reflect the ECM’s detailed composition and spatial organization [95]. Current ECM scaffold preparation techniques encompass natural polymeric hydrogel fabrication [98], collagen self-aggregation [99], 3D printing, and tissue decellularization [100]. Decellularized ECM scaffolds stand out due to their structural and compositional resemblance to *in vivo* ECMs, offering significant advantages in biomimicry [100].

Tissue decellularization emerges as a pivotal and effective strategy for creating biomimetic ECM scaffolds, aiming to closely replicate the ECM’s function and architecture to optimize cell response and tissue regeneration. Decellularized porcine hearts have been used as acellular matrices for inhabiting cells useful to engineer parts or whole organs [101,102]. Organ-on-a-chip systems have been developed to mimic tissue- and organ-level functions on chips *in vitro* [103].

Advancing tissue engineering *via* 3D cell culture hinges on the discovery of appropriate biomaterials that support the proliferation of seeded cells [104]. For this purpose, substrates have been developed through chemical synthesis, including hydrogel-based scaffolds, collagen, polycaprolactone, bacterial cellulose, and nanofibers for 3D cultures [105]. These methods are crucial for creating organoids for drug screening and autologous tissue grafts, but they face challenges due to high costs, complex multi-stage production, and difficulty in achieving precise scaffold resolution. In contrast, plant structures offer a “green” solution, providing a suitable framework for cultivating human cells *in vitro*. Using plant structures for 3D cultures could simplify the preparation process required for synthetic materials. The acellular plant framework has significant advantages over animal-based biomaterials, being economically viable and environmentally friendly.

Enhancing conventional cell culture involves replicating tissues’ physical and biochemical characteristics *in vivo*, which entails recreating cell alignment and orientation to develop functional units. These factors influence gene expression, cell proliferation, and differentiation. To achieve this, structural, mechanical, and chemical cues have been incorporated into engineered biomaterial substrates, mirroring the surface microtopography of the ECM *in vivo* [106].

Grafts and implants derived from animal and human tissues, while closely matching the physiological environment, face limitations due to their high cost and potential for immunological responses or disease transmission. This has led to increased interest in using plants as alternative sources for tissue scaffolds, recognizing their advantages in terms of availability, cost-effectiveness, and reduced risk of immune rejection or disease transmission [107].

Phytogenic polymers like cellulose [108] and pectin [109] are used to prepare soft, artificial skin. Pectin is considered as “a miracle biopolymer” due to its excellent biocompatibility, biodegradability, and antioxidant properties [110], features that are exploited in the food industry and in various bio-applications. Pectin films are transparent, soft, flexible, and thermally sensitive, and they are used in the development of artificial skin.

The diverse structural properties and surface topographies of plants, combined with their cost-effectiveness, ease of production, and modification, make them promising alternatives for tissue engineering scaffolds and biomaterials. Their inherent vascular structure, interconnected porosity, unique water transport, and retention capabilities, along with diverse mechanical properties, facilitate integration with native tissue and the restoration of cellular functionality at the injury site. The native vasculature of plants addresses the challenge of neovascularization post-implantation, as xylem and phloem channels support hydraulic conductance, enhancing the scaffold’s water transport and retention abilities. Additionally, plant surface topography and porosity create an optimal microenvironment for cell adhesion and proliferation, with patterns like microgrooves guiding cell elongation effectively [111,112,113].

### 3.1. Decellularization Process

A novel approach in scaffold preparation is the decellularization process, which allows for the direct use of plant or animal tissues as scaffolds without extracting natural polymers [114]. This method involves removing cellular content from a plant section, leaving behind the extracellular matrix structure [115]. Cells can then be seeded directly into this decellularized matrix, where they can adhere, grow, and proliferate [116]. This technique offers significant advantages, including reduced production costs associated with polymer extraction and enhanced biocompatibility and cell adhesion, given the scaffold’s natural origin and inherent cellular interaction sites [117].

While decellularized animal tissues are commonly categorized as decellularized ECMs, decellularized plant tissues are often referred to as 3D scaffolds due to differences in their composition resulting from the decellularization process.

The key distinction between animal and plant tissue decellularization lies in eliminating proteins and removing DNA content in plants following the decellularization process. In plants, since the primary component of the cell wall is cellulose, decellularized plants are regarded as an alternative source for cellulosic materials [118,119,120]. Cellulose, comprised of glucan chains with D-glucopyranose ring units linked by β(1–4) glycosidic bonds, stands as the most abundant polymer in nature, found not only in plant cell walls but also in tunicates, algae, and bacteria [121].

Cellulose-based biomaterials have garnered widespread use in various biomedical applications, including skin tissue engineering, wound dressings, bone tissue scaffolds, neural applications, and blood vessel repairs, owing to their biocompatibility and abundance. Furthermore, cellulose polymers offer adjustable mechanical properties, providing both strength and flexibility, attributed to their robust intramolecular hydrogen bonds and weak intermolecular forces [108,111].

**Figure 10:**
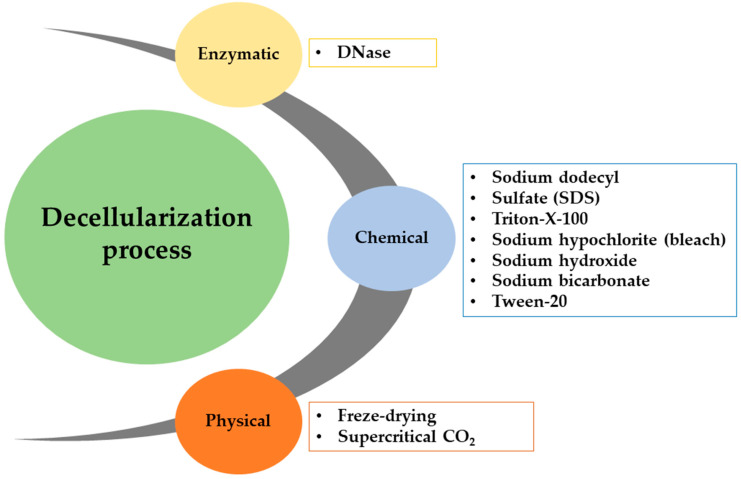
Plant decellularization methods and their commonly used chemicals.

Decellularization methods can generally be categorized into three main types, chemical, physical, and enzymatic, as illustrated in Figure 10. Table 1 provides a concise overview of the advantages and disadvantages of different decellularization methods commonly used for plant tissues. Selecting a suitable approach for decellularizing plant tissues depends on the specific application, necessitating a thorough investigation of the tissue’s structural properties and surface architecture.

***Chemical-based decellularization*** of plant tissues is widely favored due to its notable efficiency and recognition as the prevailing standard within the field. Among the diverse chemical approaches, ionic and nonionic detergent-based solutions, including SDS and Triton X-100, are frequently employed. These surfactants exert their decellularizing effects by selectively targeting lipid–lipid and lipid–protein interactions, effectively disrupting and lysing cell membranes [113]. Two primary approaches are commonly discussed in the scholarly literature: perfusion and soaking. Perfusion decellularization, particularly prevalent in studies exploring vascularization, involves the connection of a pump module containing decellularizing agents to the stalk of a plant leaf, enabling the perfusion of the decellularization solution through the veins. Conversely, soaking-based decellularization, a more widespread method, entails immersing the entire tissue in a solution to achieve comprehensive tissue decellularization [124].

Freeze-drying and supercritical CO_2_ treatment are recognized as ***physical plant decellularization*** methods [125,126], albeit less commonly utilized compared to chemical approaches. Even after thorough washing procedures, chemical methods, notably employing detergents like SDS and Triton X-100, may leave residual chemical traces. The primary advantage of opting for physical decellularization methods is preserving 3D scaffolds devoid of surfactants, thereby ensuring the retention of their inherent biocompatibility.

***Enzymatic decellularization*** is commonly used for cell removal and is typically combined with chemical decellularization to improve cell removal and fully break down nuclear components in tissues [127]. Commonly used enzymes for tissue decellularization are trypsin, nucleases, collagenase, lipase, and dispase [128]. Trypsin is a proteolytic enzyme that decellularizes cardiac valves and the dermis [129]. Trypsin’s impact on peptides limits its use on extracellular components like collagen and elastin, due to the potential for undesired alterations to the ECM [130]. Trypsin treatment can decrease the processing time and be combined with other chelating and/or chemical agents to avoid harm to collagen and elastic fibers. Nucleases act as enzymatic decellularizing agents by cleaving DNA and RNA strands using DNase and RNase, respectively. Collagenase and dispase are enzymes that can produce decellularization. Enzymatic agents are more effective when combined with chemical, physical, or chelating agents rather than on their own. Apart from *in vitro* investigations, enzymatic approaches have remained relatively underexplored due to the continual advancements in chemical and physical decellularization methodologies. To the authors’ knowledge, only one study has utilized an enzyme as the primary decellularization agent. In this study, transgenic hairy roots derived from tobacco (*N. tabacum*) were subjected to decellularization using a combination of permeabilization achieved through freeze-drying and treatment with DNase. Subsequent analysis revealed a significant reduction in nuclear content post-decellularization compared to control samples [119]. Although the published results present promise, further exploration and investigation of enzymatic approaches are warranted to enable meaningful comparisons against conventional methods.

### 3.2. Applications of Decellularized Plants in Bone Tissue Engineering

**Figure 11:**
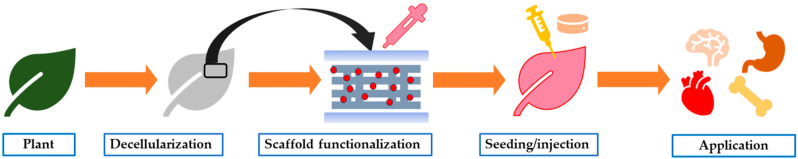
Applications of decellularized plants in tissue engineering.

In recent years, the utilization of decellularized plant tissues has emerged as a novel approach for creating cellulose-based scaffolds for three-dimensional (3D) cell culture applications. Given the extensive diversity within the plant kingdom, materials derived from plants offer a wide array of structural configurations, including voluminous frameworks, branching systems, and vascular channels, thereby facilitating the tailored selection of designs to match the specific architectural needs of engineered tissues (Figure 11). Leveraging their inherent characteristics, structures obtained from sources such as apples, onions, leeks, and carrots have been employed to meet precise porosity and surface criteria [120,131]. Conversely, stems and natural venation materials from plants like spinach and bamboo are favored for forming vascular networks [114].

The ECM in plants is characterized by an intricate array of molecules that envelop and bolster plant cells. This matrix encompasses various proteins, carbohydrates, and additional compounds that impart structural stability and influence cellular functions. It is instrumental in mediating plant development, growth, and adaptability to environmental changes. The heterogeneity inherent in plant life furnishes a spectrum of structural, mechanical, and biochemical attributes, thereby augmenting their potential to emulate the properties of human tissues and their capacity for biomimicry.

To enhance the utility of plant-based scaffolds for tissue engineering applications, these scaffolds are subjected to surface functionalization techniques and cell seeding processes to improve cellular adhesion and promote cell survival. Notably, the ECM derived from plants inherently lacks certain proteins in mammalian tissues, such as proteoglycans and fibrous proteins like collagen and fibrin. These are essential for creating an environment conducive to cell growth and are subsequently crucial for tissue metabolism [132]. To address this limitation, strategies such as protein-based surface biofunctionalization and chemical alterations of deproteinized plant tissues are applied to cellulose scaffolds. These modifications facilitate better cell attachment and enhanced cell adhesion. While previous research has indicated that biofunctionalization is not required for the maturation of cells on plant-based scaffolds [133,134], applying ECM protein coatings, including fibronectin, collagen, and RGD peptides, remains a prevalent approach to foster cell–scaffold interactions and surface adhesion [120,134,135,136,137]. Specifically, the RGD sequence is known for its cell-binding capabilities, and natural proteins like collagen and fibronectin are major constituents of the native ECM [138]. For example, Lacombe et al., applied a collagen and fibrin coating mix to spinach leaves before seeding cells, notably facilitating the attachment of melanoma and prostate cells [135]. Poly-L-lysine, chitosan, gelatin, and human ECM proteins have been utilized to achieve similar objectives [131,136].

Conversely, surface chemical modifications are viable strategies to augment tissue regeneration capabilities. Specifically, amyloid aggregation and silanization have been identified as effective surface treatments for plant-based materials [139,140]. In greater detail, amyloids, which are aggregates of proteins, have been shown to facilitate cell adhesion and promote nano-hydroxyapatite deposition, thereby supporting bone regeneration processes [139]. Similarly, the covalent bonds formed during organosilanization can foster hydrophobic and electrostatic interactions with cellular entities [140]. Various cell types, including human-induced pluripotent stem cell-derived cardiomyocytes (hiPSC-CMs), human umbilical vein endothelial cells (HUVECs), and osteoblast precursors, among others such as human dermal fibroblasts (HDFs), human dermal microvascular endothelial cells (HDMECs), and human breast cancer cell lines, can repopulate acellular plant tissues tailored for specific tissue engineering applications [134,139,140,141,142,143].

Cell seeding on these scaffolds, derived from either plant leaves or stems [144], can be directly executed. The cellulose structure inherent in plant tissues provides an optimal foundation for mammalian cell adhesion and proliferation. By engineering the surface microtopography of decellularized plant tissues to mimic *in vivo* ECM, complete with customized mechanical and chemical attributes, seeded cells can proliferate and organize within the biomaterial, emulating tissue-specific physiological architectures (e.g., cellular orientation and structural configurations) [114,145]. To construct more sophisticated scaffolds, techniques combining surface cell seeding and injection have been developed [118,139], yielding constructs that more closely resemble natural tissues. The pre-existing vascular networks in plants offer a blueprint for designing perfusable vascularized tissues, with injection methods facilitated *via* cannulation of the primary stem, enhancing the prospects for *in vivo* implantation through pre-cellularization, which contributes to tissue neovascularization and colonization.

The innate vascular structures of certain plants, such as spinach, baby sun rose, and parsley, which support transport functions within the plant, are leveraged in tissue engineering to develop vascularized tissue scaffolds. The engineering of such branched architectures facilitates essential biological functions like nutrient and gas exchange and waste elimination within the tissue constructs, presenting a significant advantage over current biofabrication technologies, such as 3D bioprinting [146]. Research into preserving these prefabricated vascular systems post-decellularization has included experiments with colored dyes (e.g., Rhodamine and Ponceau Red) to verify the functionality of the vascular network and ensure diffusive capacity to microvessels [147,148]. Cell injection techniques are further utilized to re-endothelialize and populate these plant-based scaffolds with a natural vascular system, aiming to replicate the hierarchical structure of mammalian vascular networks [142,148]. While manual syringe injections can introduce variability in flow rates and potentially damage the scaffold, programmable syringe pumps offer a solution by providing a controlled dynamic microenvironment and preserving the structural integrity of the cellulose-based architecture [126,149,150].

Bone constitutes a mineralized connective tissue essential for providing structural support and facilitating the body’s mechanical functions. It serves as a protective layer for crucial organs, with its architectural characteristics varying with age and specific body regions [151]. Owing to its complex heterogeneity, encompassing both an organic phase (proteins like collagen) and an inorganic matrix (predominantly hydroxyapatite (HA)) and its limited self-repair capability for critical-sized defects (<2 cm), current bone tissue engineering (BTE) strategies focus on developing scaffolds that emulate bone’s native architecture and biological functionalities [152,153]. These strategies aim to synthesize materials which replicate bone’s composition while ensuring biocompatibility, biodegradability, and regenerative capacity [154]. Traditionally, single-phase materials such as polymers (both natural and synthetic), ceramics, and metals have been extensively utilized. Metals, including Mg and Ti and their alloys, are preferred for their durability and the ability to fabricate scaffolds with controlled porosity to support load-bearing functions. Conversely, bioceramics, like calcium phosphates (including HA and tricalcium phosphate or TCP), more closely resemble bone’s inorganic component and can better facilitate osteo-differentiation [155,156]. Natural polymers (e.g., collagen, alginate, and gelatin) are adept at mimicking the ECM and offer superior biological properties. Synthetic polymers (e.g., PCL, PLA, PGA, and PLGA) provide enhanced control over scaffold porosity and mechanical properties, though they generally exhibit lower cytocompatibility [157]. Recently, composite materials have started to be recognized for their potential to improve bioactivity and mechanical properties of bone-like scaffolds, with various additives or fillers (e.g., antibiotics, reduced graphene oxide, and nanosized HA) being integrated into the primary matrix to bolster material properties [158,159]. Yet, challenges persist in biomaterial–tissue engineering (BTE) regarding material–host tissue interactions (e.g., immunogenicity and antimicrobial attributes), vascularization strategies, and the controlled release of physicochemical stimuli to optimize osseointegration and osteogenesis [160].

When creating plant-based scaffolds for BTE, it is crucial to consider the mechanical strengths and fatigue resistance of the plant materials used. These properties are essential to ensure that the scaffolds can withstand the physical stresses and loads that bone tissues typically experience in the body. Choosing plants with suitable mechanical characteristics helps to maximize the scaffold’s effectiveness and durability, promoting better integration and functionality in bone repair and regeneration. The mechanical properties of plants, such as strength, durability, and elasticity, vary widely. For instance, *Apple hypanthium* tissue has a Young’s Modulus between 1 and 4 kPa, making it suitable for soft tissue applications, while palm fibers, with a Young’s Modulus as high as 4 GPa, are appropriate for bone tissue scaffolding [122]. However, this variability can also pose challenges, as different tissues require specific properties like fatigue resistance for bone scaffolds. These factors must be taken into account. The current literature, including studies by Iravani and Varma [114] and Bilirgen et al. [113], indicates that more research is needed to confirm if the mechanical characteristics of plants meet the requirements for effective tissue engineering, especially in *in vivo* environments.

Distinct plants possess unique architectures and morphological traits, offering opportunities for creating adaptable constructs (Table 2).

Typically, plant-derived structures exhibit mechanical properties inferior to native bone tissue (elastic modulus, E = 3.5–19 GPa), positioning them primarily as candidates for non-load bearing bone fillers. Nonetheless, the mechanical attributes of such engineered scaffolds may improve over time through cellular deposition of inorganic ECM components, *in vitro* or *in vivo*, thus narrowing the mechanical disparity between the scaffold and native tissue [161]. To replicate bone’s structure, cellulose scaffolds often undergo functional coatings and surface modifications to enhance cell growth and mechanical strength. For example, nano amyloid/hydroxyapatite-coated decellularized spinach leaves significantly improved osteoblast-like MC3T3-E1 cell adhesion, compared to untreated decellularized plant tissues (dPT) [161]. This microstructural guidance of cells, coupled with biofunctionalization, not only facilitated cell distribution but also markedly increased the Young’s Modulus (coated—max 60 kPa vs. pristine—20 kPa). Endothelial cells (HUVECs) and myoblast precursors (C2C12) seeded on spinach stem surfaces treated only with nano-amyloid, forming a dual-layer tube-like structure, hint at potential applications beyond bone regeneration, such as in vascularized muscle tissues or more complex structures like bone marrow.

Additionally, poly-L-lysine coatings have been shown to enhance pre-osteoblast adhesion on carrot-derived scaffolds, despite these constructs exhibiting a compression modulus significantly lower than that of natural bone, suggesting their suitability for non-load bearing applications. The authors of this work analyzed apples, carrots, and celery, finding that these remained porous, with sizes between 70 and 420 µm following decellularization. Based on their pore sizes and mechanical properties, these fruits and vegetables were recommended as potential scaffolds for fat, bone, and tendon tissues, respectively, due to their support for cell growth and differentiation [131]. Moreover, surface modifications with organosilanes on *Asian palmyra* palm immature endosperms, characterized by a fibrous interconnected structure, enhanced MG63 osteoblast differentiation and mineralization, endorsing the osteoinductive potential of these functionalized scaffolds, also achieving a superior compressive modulus (coated—8.33 ± 1.52 kPa vs. pristine—0.59 ± 0.19 kPa), suggesting their utility as a bone filler material in both non-load bearing and soft tissue bone regeneration contexts [140].

Enhanced mechanical performance was also observed in decellularized *Alstroemeria* flower stem scaffolds coated with chitosan, which showed an increased compressive modulus (0.8 MPa vs. non-coated 0.15 MPa, respectively), elasticity, and elongation compared to non-coated scaffolds, indicating their beneficial role in cell attachment [147]. The chitosan coating further facilitated cell adhesion and the creation of perfusable BTE constructs, leveraging the cylindrical geometry and internal native microchannels, as the analysis demonstrated a rough surface texture conducive to cell adherence on the treated structures [162]. Consequently, such treated stems were seeded with MC3T3 cells, displaying low cytotoxicity levels. Various hierarchically organized plant-derived scaffolds, including bamboo stems, spinach leaves, and onions, have been explored for fabricating bone-like constructs, emphasizing inducing osteogenic differentiation in mesenchymal stem cells [163,164]. For instance, common bamboo (*Bambusa vulgaris*), selected for its structural resemblance to native bone and its composite nature, underwent decellularization and modification *via* a sodium periodate-based oxidation process, enhancing the biodegradation and biocompatibility of scaffolds. However, this treatment reduced cellulose scaffold crystallinity, resulting in decreased compressive strength, aligning with the mechanical range of cancellous bone (1.52 MPa, 0.5–20 MPa), thereby indicating their potential for craniofacial and maxillofacial regeneration (non-load bearing applications). Following treatment, mesenchymal stem cells were seeded on the scaffolds, showing osteogenic differentiation after 24 h. Macrophage infiltration and blood vessel formation in *in vivo* subcutaneous implants in Wistar rats at four weeks post-implantation were observed, both peripherally and centrally, contrasting with partial infiltration in non-oxidized samples.

A novel application leverages the unique structural properties of onion cells, such as their layered architecture, which could be engineered to contract and expand in response to certain stimuli, mimicking the behavior of muscle fibers [165]. The research group of Chen developed an actuator made from botanic epidermal cells [166]. A freeze-drying treatment was employed, effectively removing water without damaging the cell structure, thus preserving the integrity and functionality of the cells for actuator applications. The freeze-drying process keeps the microstructure of the onion epidermal cells intact and makes them stiff and brittle. An acid pretreatment process was utilized to remove the hemicellulose from the cell wall to make the dried cells elastic. After the acid pretreatment, only a small amount of hemicellulose remained in the cell wall of the onion epidermal cells. At voltages of 0–50 V, the onion artificial muscle elongated and bent downward, and at 50–1000 V, the artificial muscles contracted and bent upward [166].

Researchers have developed artificial muscles mimicking the tropisms of helical plants, capable of self-adapting to environmental changes in humidity and temperature. These muscles, made from hierarchically patterned helically wound yarns inlaid with hydrogel, showcase rapid movement and large strokes, embodying plant-like microstructural memories for autonomous operation in real-life applications [167].

### 3.3. Challenges of Decellularized Plant Scaffolds for Transplantation

Even though decellularized scaffolds have garnered considerable interest in tissue engineering and transplantation due to their potential to mimic the ECM, several challenges can impede their successful application.

One significant issue is the incomplete removal of cellular components during the decellularization process. Despite using chemical, physical, or enzymatic methods, residual DNA and cellular debris can remain, potentially provoking immune responses in the recipient. Preserving the integrity of the ECM during decellularization is another major hurdle. The process can sometimes compromise the structural and functional properties of the ECM, which are crucial for supporting cell adhesion, proliferation, and differentiation. This damage can impair the scaffold’s ability to recreate the natural tissue environment effectively.

Matching the mechanical properties of the scaffold with those of the native tissue is also challenging. For example, plant-derived scaffolds may not have the necessary mechanical strength and durability required for load-bearing applications in bone tissue engineering. Additionally, achieving efficient recellularization, where new cells are seeded onto the decellularized scaffold, requires optimal conditions to promote cell adhesion, growth, and differentiation. Ensuring uniform cell distribution and maintaining cell viability throughout the scaffold are significant obstacles.

Immune response remains a concern even with thorough decellularization. The recipient’s immune system may still recognize the scaffold as foreign, leading to an immune reaction, particularly with scaffolds derived from animal sources. Another critical issue is the development of an adequate vascular network within the scaffold to supply nutrients and oxygen to the seeded cells. Without proper vascularization, cells can die, leading to the failure of the tissue graft.

The lack of standardized protocols for decellularization and recellularization processes contributes to variability in scaffold quality and performance, hampering reproducibility and scalability for clinical applications. Furthermore, the processes involved are often time-consuming and costly, posing challenges for developing cost-effective and scalable methods for widespread clinical use. In conclusion, while decellularized scaffolds hold significant promise for tissue engineering and regenerative medicine, their successful application faces several challenges.

Addressing issues related to decellularization efficiency, ECM preservation, mechanical properties, recellularization, immune responses, vascularization, standardization, and cost is critical for advancing their use in clinical settings.

### 3.4. Applications of Decellularized Plants in Drug Testing

Decellularized spinach leaves were used to develop a bioinspired *in vitro* model for nanopharmaceutical drug testing. A thrombosis has been recreated in the spinach leaf’s vasculature of this model for testing the effect of both a single-chain urokinase plasminogen activator (scuPA) and a magnetically controlled nanocomposite prepared by the heparin-mediated cross-linking of scuPA with Fe_3_O_4_ nanoparticles [168]. This is an eco-friendly approach to replace animal testing.

A recent study reported the preparation of a new cellulosic 3D scaffold based on decellularized tomato thorny leaves which was recellularized with human hepatocellular carcinoma cancer cells (HepG2 cells) to mimic the tumor microenvironment for chemotherapeutic testing of prilocaine, an anticancer drug, on hepatocellular carcinoma [169]. This vegetal scaffold is an ideal candidate for liver cancer modeling.

## 4. Plants in Green Electronics. Bionic Light-Emitting Plants

### 4.1. Plants in Dye-Sensitized Solar Cells (DSSCs)

DSSCs represent a significant advancement in photovoltaic technology, utilizing dye molecules to enhance the performance of a metallic oxide (semiconductor) photoanode. These solar cells are inspired by the natural process of photosynthesis, effectively converting sunlight into electrical energy. A DSSC comprises several crucial components: the photoanode, sensitizer, electrolyte, and counter electrode. The photoanode is crafted from semiconductor nanostructures such as nanorods, nanowires, nanotubes, nanocones, and nanosheets. These are synthesized on conductive glass, which can be either transparent or opaque, to form the backbone of the cell [170]. The unique properties of these nanostructures, including their increased surface area, facilitate enhanced light absorption and electron transport, crucial for the cell’s efficiency [171].

The fundamental components of a conventional DSSC encompass a transparent conductive oxide (TCO), a dye sensitizer, a semiconductor oxide, a counter electrode, and an electrolyte. Within this configuration, a nanoporous semiconductor oxide, underpinned by conductive glass, serves as the working electrode. This layer is separated from the counter electrode by a minimal layer of electrolyte solution, which facilitates electron transport. By extending the coverage of the dye over the photoelectrodes, it becomes possible to capture lower-energy photons, enhancing light absorption. The dye molecules chemically bond to the semiconductor’s oxide surface, ensuring efficient energy transfer [172].

For optimal functionality, the sensitizer must exhibit a robust affinity for the semiconductor oxide, enabling the absorption of a broad spectrum of wavelengths while retaining thermal stability. The photoanode of the DSSC is typically composed of a thin film (approximately 10 µm) of TiO_2_, though nanoparticles of SnO_2_ or ZnO may also be used less frequently. The extensive surface area provided by the TiO_2_ layer is crucial for effective light absorption [173].

The achievement of superior photoelectrochemical output and maximal conversion efficiency necessitates detailed consideration of the material bandgaps and the structural arrangements of each layer within the fabrication process of the DSSCs. These considerations are essential for optimizing the device’s efficiency and operational stability.

**Figure 12:**
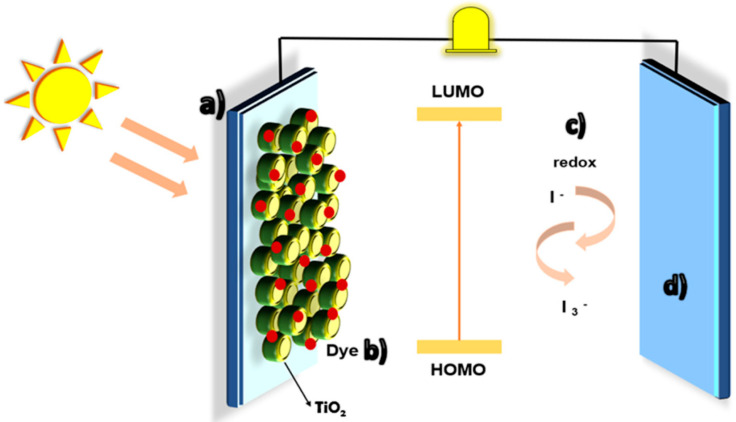
Conventional architecture of DSSCs [174].

The conventional architecture of DSSCs, as depicted in Figure 12, employs a “sandwich” configuration composed of four principal components:

*a) Light-sensitizing dye*: This component involves dye molecules that are integrated with the semiconductor material to form the photoanode. The dye is crucial for absorbing sunlight and facilitating the initial charge separation by generating excited electrons.

*b) Electrolyte*: The electrolyte contains a redox couple, typically iodide/triiodide, which plays a vital role in the cell’s operation. It acts as a medium for the transfer of electrons between the photoanode and the counter electrode, helping to regenerate the dye after it has donated electrons.

*c) Counter electrode*: Generally coated with platinum or carbon graphite, the counter electrode functions to collect electrons from the external circuit and facilitate their transfer back into the electrolyte, thus completing the electrical loop.

*d) Photoanode*: The photoanode consists of a semiconductor oxide, often titanium dioxide (TiO_2_), deposited on a transparent substrate. This layer provides a vast surface area for the adsorption of the dye molecules and is instrumental in the effective conversion of light into electrical energy.

The operational principle of a solar cell hinges on the photon captures by the dye, which necessitates that the sensitizing molecule exhibit significant absorption in the visible region of the electromagnetic spectrum, where solar radiation is most intense. Upon exposure to sunlight, the dye, typically adsorbed onto a photoelectrode made of nanostructured TiO_2_, undergoes excitation. This excitation involves the promotion of an electron from the dye’s highest occupied molecular orbital (HOMO) to its lowest unoccupied molecular orbital (LUMO). The excited electron is subsequently transferred to the mesoporous TiO_2_ conduction band. TiO_2_’s extensive surface area facilitates injecting a substantial volume of charge carriers.

The electron moves to the transparent conductive oxide and is channeled through an external circuit to the counter electrode. Here, the electron is conveyed to the electrolyte, which then returns it to the dye, effectively regenerating it. This regeneration allows the dye to absorb another photon and reinitiate the cycle, thus perpetuating the process of energy conversion [175,176,177].

Separated charges in artificial photosynthesis enable water oxidation and carbon dioxide reduction. Water oxidation produces oxygen gas and protons, while CO_2_ reduction converts CO_2_ into compounds like carbon monoxide, formic acid, methanol, or methane. The efficiency of these reactions depends on the catalysts’ activity, selectivity, and stability. Redox mediators facilitate electron transport, reduce energy loss, and prevent charge recombination, influencing CO_2_ reduction selectivity. The final stage produces desired products such as hydrogen gas and fuels, with product distribution affected by catalytic thermodynamics, kinetics, and reactant concentrations. Membrane separators or gas-diffusion electrodes can enhance efficiency by separating products [178].

Photochemical cells initiate artificial photosynthesis by absorbing light with a photosensitizer, a light-absorbing substance that produces excited electrons when illuminated. The photosensitizer can be an organic, inorganic, or quantum dot, each possessing distinct light absorption properties [179]. The photosensitizer’s efficiency is influenced by its capacity to absorb a wide range of the solar spectrum and its excited state lifespan, which impacts the charge separation process. When light is absorbed, the energized electrons move from the photosensitizers to an appropriate electron acceptor, while the positive charges (holes) are transferred to an electron donor. The charge separation mechanism is crucial for transforming absorbed light energy into chemical energy and preventing the quick recombination of charges, which could lead to energy loss [179].

Organic dyes, including metalloporphyrins, phthalocyanins, and ruthenium polypyridyl complexes, are commonly used in DSSCs due to their potent absorption and high molar extinction coefficients. Mathew and colleagues conducted a study using a molecularly designed porphyrin dye called SM315 in DSSCs [180] (p. 13). This dye has a donor–π-bridge–acceptor structure that enhances electrolyte compatibility and light-harvesting capabilities. Using SM315 with the cobalt (II/III) redox shuttle led to the development of dye-sensitized solar cells with a high open-circuit voltage (VOC) of 0.91 V, short-circuit current density (JSC) of 18.1 mA cm^–2^, fill factor of 0.78, and a power conversion efficiency of 13% [181,182]. Despite being cost-effective and having adjustable absorption qualities, organic dyes face hurdles due to their poor light-harvesting efficiency and long-term stability. Recent advancements in molecular engineering have led to the creation of novel organic dyes that exhibit enhanced performance and stability [183,184].

Inorganic dyes like cadmium sulfide (CdS) and cadmium selenide (CdSe) are sensitizers because of their increased stability and broader absorption spectra than organic dyes. However, their toxicity and possible environmental effects are significant issues. Perovskite materials, known for their significant efficiency enhancements in solar cells, can also be classified as inorganic dyes and have shown potential as materials [185]. Yoo and colleagues [186] utilized a holistic method to enhance the performance of perovskite solar cells by improving charge carrier management. Initially, they created an electron transport layer with specific film coverage, thickness, and composition by adjusting the chemical bath deposition of tin dioxide (SnO_2_)—the passivation technique for the bulk and the interface, enhancing characteristics and reducing the bandgap penalty. The devices demonstrated an electroluminescence external quantum efficiency of 17.2% and an electroluminescence energy conversion efficiency of 21.6%. The solar cells reached a verified power conversion efficiency of 25.2%, which is 80.5% of the thermodynamic limit of their bandgap [187].

Quantum dots, also known as semiconductor nanocrystals, are considered effective sensitizers for artificial photosynthesis because of their distinctive optical characteristics, including a bandgap that can be adjusted in size and generate numerous excitons. They have demonstrated enhanced efficiency compared to organic dyes. However, their toxicity and possible environmental consequences are still significant concerns. Recent research has concentrated on creating alternative quantum dot materials with lower toxicity, such as copper indium sulfide (CIS) [188] and silver indium sulfide (AgInS_2_) [189].

### 4.2. Insights into Natural Pigments for DSSCs

The demand for sustainable energy solutions has intensified research into advanced technologies, highlighting DSSCs as a notable development in renewable energy. Central to the functionality of DSSCs is the choice of sensitizers, which are crucial for optimizing both efficiency and environmental sustainability [190]. Natural dyes have emerged as favorable sensitizers due to their renewable nature and minimal environmental impact. Distinguished by their cost-effectiveness and reduced environmental and health impacts, DSSCs offer a simpler and more economical manufacturing process compared to traditional photovoltaics, achieving efficiencies over 14% [180].

Natural dyes derived from leaves, flowers, and fruit peels are particularly promising for DSSCs. Yet, their performance depends on factors such as charge collection efficiency, photo-scattering properties, and recombination rates [191]. Despite their promise, natural dye-based DSSCs still lag behind conventional photovoltaic technologies like silicon solar cells in terms of efficiency. Historically, before synthetic dyes were introduced in 1856, natural dyes from plants, minerals, animals, and microbes were the primary color sources [192]. Predominantly derived from plants, these pigments were used not only in textiles but also in food, medicine, crafts, and other applications, underscoring their renewable and biodegradable advantages.

Contemporary research in DSSCs explores various natural compounds as sensitizers, including cyanins, anthocyanins, tannins, chlorophylls, betalains, and carotenoids. Anthocyanins are particularly notable for their broad visible spectrum absorption, attributed to charge transfer transitions, making them effective for use in various plant tissues [193].

Research underscores the viability of natural dyes as sensitizers in DSSCs, highlighting the ecological benefits over synthetic and metal-based dyes [194]. Insights from photosynthesis have demonstrated the crucial role of natural pigments in solar energy absorption, advocating for their use in solar energy conversion. Chlorophyll, as the primary plant pigment with a high visible light absorption coefficient, alongside supplementary pigments, enhances the light absorption range, crucial for the energy-producing processes of photosynthetic organisms [195].

The simplicity of extracting natural dyes from plants and their low processing requirements promote the development of cost-effective, environmentally friendly DSSC systems. Natural dyes offer several advantages over synthetic alternatives, including easier extraction, application without intensive purification, reduced environmental impact, and lower production costs [195].

The extraction of natural dyes for DSSCs entails a meticulous procedure aimed at harnessing pigments from botanical sources, critical for achieving optimal solar cell performance. This process begins with the careful selection of plant materials known for their vibrant colors, including leaves, flowers, and fruit peels. Following the thorough cleaning and drying of these materials, various extraction methods are applied. Techniques such as maceration or heat-assisted extraction are commonly used to effectively release the natural pigments. Subsequent purification steps, including filtration or chromatography, are essential to isolate the desired dyes from other compounds extracted concurrently. This purified natural dye is then subject to detailed chemical characterization to determine its molecular structure, composition, and optical properties. Such information is crucial for optimizing the dye-sensitized layer within DSSCs, particularly in adjusting factors like the concentration of the dye, the thickness of the film, and the duration of adsorption. These adjustments are key to enhancing the light-harvesting capabilities and electron injection efficiency of the solar cells. Ultimately, the process of extracting natural dyes underscores the environmentally conscious approach of DSSCs, positioning them as a sustainable alternative to synthetic dyes in the field of renewable energy applications [196].

Dyes utilized in the fabrication of DSSCs are categorized into organic (natural) dyes and inorganic dyes. Among inorganic dyes, ruthenium (Ru)-based compounds are prominent due to their role in enhancing the efficiency of DSSCs. However, these dyes are costly and pose challenges in terms of purification. In the quest for more sustainable and cost-effective alternatives, natural dyes have emerged as favorable substitutes due to their lower production costs, ease of manufacturing, short payback periods, flexible usage, readily available raw materials, and minimal environmental risks [197].

Natural pigments align with environmental protection standards and exhibit superior performance under diffuse and multicolored light conditions. Over recent years, various plant parts such as leaves, petals, and bark have been explored for their potential as sensitizers. The unique properties and parameters of these natural pigments have resulted in varying levels of efficiency in DSSCs [198].

Plant pigmentation is influenced by the specific structures of pigments that adapt to sunlight exposure, altering the absorption wavelengths. These pigments typically possess a broad absorption spectrum within the visible region, contributing significantly to the performance metrics of DSSCs such as the fill factor, energy conversion efficiency, open-circuit voltage, and short-circuit current. Variations in natural dyes derived from different plant parts have shown diverse effects on the performance of these dyes as photosensitizers for DSSCs [199].

The most common pigments are betalains, carotenoids, chlorophyll (Chl), and flavonoids such as anthocyanins.

Chlorophyll represents the green pigment prevalent in the foliage of most plants, cyanobacteria, and algae. Among the various shades of Chl-based dyes, chlorophyll *a* (Chla) is the most ubiquitous. Structurally, chlorophyll is a chelate, comprising hydrogen, carbon, a sizable metal ion that connects to significant molecules, and other elements like oxygen and nitrogen. In the photosynthetic process, Chl absorbs energy, facilitating the transformation of carbon dioxide into carbohydrates and splitting water into oxygen, thus converting solar energy into a usable form for plants. The molecular configuration of chlorophyll includes a chlorin ring centered around magnesium, adorned with various side chains and small hydrocarbons, which vary according to the type of Chl [200]. In the case of image processing systems, Chl is paramount, primarily functioning to harvest sunlight, transform solar energy into chemical forms, and facilitate electron transitions. Chl encompasses a spectrum of over fifty pigments that include chromophores. Due to their efficient light-harvesting capabilities, Chl and its derivatives are integrated into DSSCs as photoanodes. Notably, Chla (chl-methyltrans-32-carboxypyroformide) is distinguished for its effectiveness. It has been documented that Chl can catalyze the performance of four semiconductors to peak activities in TiO_2_ and ZnO within diverse settings. At a wavelength of 670 nm, Chl exhibits moderate absorption, thereby acting effectively in the visible spectrum [199]. Chl a, the principal pigment in plant tissues, is composed of C_55_H_72_O_5_N_4_Mg and displays significant absorption peaks at 430 nm and 662 nm, presenting vivid green and blue hues. In contrast, chlorophyll b, with a chemical formula of C_55_H_72_O_5_N_4_Mg, features a structural variation where methyl groups are replaced by CHO groups, and it appears bright blue with absorption peaks at 453 nm and 642 nm [200]. Chla is a valuable photopigment which strongly absorbs in the blue and red region of the electromagnetic spectrum, and it also presents a strong emission peak in the red region when exciting in the Soret band. These spectral features have been exploited in various biophysical studies. Thus, Chla inserted in biomimetic membranes was used as an optical sensor to monitor the effects of different physical, chemical, or pharmacological agents on artificial cell membranes, at a molecular level [201,202]. Moreover, Chla was also used as a spectral marker to provide deep insight into the formation of the biohybrids containing Chla-loaded biomimetic membranes [40,203].

Flavonoids are a diverse group of plant pigments, with over 5000 natural variants. They include flavonoids, flavones, flavanones, isoflavones, catechins, anthocyanins, and chalcones, and they are categorized by their chemical structures: flavonoids (2-phenyl benzopyrans), isoflavonoids (3-benzopyrans), and neoflavonoids (4-benzopyrans). Typically, flavonoids have a 15-carbon skeleton with two phenyl rings connected by three carbon bridges, forming an additional ring. Their oxidation level determines their color, and not all can absorb visible light. Those that have free electrons enable visible light absorption. Ecologically, flavonoids attract animals for seed dispersal and insects for pollination, with many absorbing UV radiation and creating patterns visible to bees. They protect plant foliage from harmful UV rays. Key subcategories are flavanols, anthocyanins, and proanthocyanidins, each contributing to plant–environment interactions through biochemical and photoprotective functions [200].

Carotenoids are organic pigments in both the chloroplasts and chromoplasts of plants, as well as in certain photosynthetic organisms, including some species of fungi and bacteria. These pigments serve two critical roles: they absorb light energy for utilization in photosynthesis and provide photoprotection to chlorophyll from light-induced damage. Carotenoids impart the characteristic red, yellow, and orange hues to many flowers and fruits and contribute to various aromas. Over 600 distinct carotenoids have been identified, categorized into carotenes, which are pure hydrocarbons, and xanthophylls, which contain oxygen. Structurally, carotenoids are tetraterpenoids, synthesized from eight isoprene units, thus comprising 40 carbon atoms. Typically, carotenoids absorb light in the wavelength range of 400 to 550 nm, corresponding to purple to green light [200,204].

### 4.3. Plants in Bioelectronics: Natural/Plant-Based DSSCs

Plants are essential to our environment and crucial for human existence. Photosynthesis is the process by which plants convert sunlight into chemical energy and control atmospheric carbon dioxide and oxygen levels [205]. This process creates a suitable environment that sustains life on Earth. Plants have a crucial part in human progress, serving as the main food source and offering a wide range of materials such as fibers for clothes [206], wood [207], fuel [208], construction [209], and paper [210], as well as valuable compounds for medicines [211] and cosmetics [212]. Furthermore, plants are crucial in promoting our overall well-being, whether found in their native environment or incorporated into urban settings. They provide a sense of tranquility, breathtaking scenery, and a connection to our natural surroundings. Plant biology research relies on advanced molecular and genetic techniques conducted in living organisms and invasive tissue sampling subsequently analyzed outside the organism [213]. A significant limitation of genetic engineering is its predominant focus on model species, often challenging its application to other species [214].

On the contrary, invasive sampling disrupts plant signaling, resulting in *ex vivo* examined samples that do not accurately represent the plant’s normal condition. Bioelectronic technology can enhance traditional methods and provide novel opportunities for monitoring and adjusting plant physiology in real time [215]. Bioelectronic sensors can convert intricate biological inputs into electronic readout signals, whereas bioelectronic actuators may manipulate biological networks through electronic addressing. Specifically, devices using organic electronic materials as active layers have the benefit of signal transduction. This is because they combine ionic and electronic conduction, allowing for close interaction with the naturally ionic biological environment [216].

Nevertheless, the bioelectronics sector is primarily propelled by the discipline of biomedicine to provide novel medicines and diagnostic tools. As a result, the application of these technologies in plants has been less extensive compared to mammals. Other factors could have contributed to this situation, including the absence of a historical or traditional foundation, the emphasis on genetic approaches in plant biology science, limited funding for research in plant science, and limited collaboration between the plant biology and electronic and engineering fields [217].

For example, chlorophylls, which are a group of pigments found in the chloroplasts of plant cells and the chloroplast-like organelles of algae [218], come in various types, with chlorophyll-a and chlorophyll-b being the most common. Their structure consists of a porphyrin ring with a magnesium ion at the center, responsible for their green color [219]. Chls play a critical role in photosynthesis by absorbing sunlight and converting it into chemical energy, making them an attractive choice for solar cell technology [220]. Chls stand out as the sole naturally occurring organic semiconductors. When harnessing Chls, the journey from a readily available source like spinach or spirulina to creating a solid-state device with conventional photovoltaic properties is likely one of the most efficient and minimalistic routes within the domain of modern optoelectronics [221,222]. Understanding the natural photosynthesis process is crucial to harnessing the potential of chlorophylls in solar cells [223]. During photosynthesis, Chl captures photons of sunlight, which excite electrons [224]. These energized electrons are transferred through reactions, producing energy in adenosine triphosphate (ATP) and reducing equivalents, such as NADPH. This natural process inspires the design of artificial photosynthetic systems in solar cell technology [225]. Chlorophyll, as the sensitizer, plays a vital role in this step. Chl molecules were immobilized on the surface of the semiconductor layer, typically titanium dioxide (TiO_2_) [226]. The unique structure of chlorophyll allows it to absorb photons effectively over a broad spectrum of visible and near-infrared light [226]. When chlorophyll molecules absorb photons, their electrons become excited and are raised to higher energy levels. These excited electrons are referred to as photoexcited electrons. The photoexcited electrons in chlorophyll are injected into the semiconductor layer (TiO_2_) [226]. This process is driven by the difference in energy levels between the excited state of chlorophyll and the conduction band of the TiO_2_ semiconductor. Once injected into the TiO_2_, the electrons are mobile and can move through the material. The injected electrons in the TiO_2_ semiconductor travel through its porous structure, facilitated by the mobility of the electrons. This movement creates an electric current within the semiconductor. As electrons are injected from chlorophyll into the semiconductor, chlorophyll molecules become positively charged (oxidized) [227]. To maintain the sensitizer’s ability to absorb more photons, the electrolyte solution within the cell regenerates the oxidized chlorophyll molecules by providing electrons [227]. The electrons collected by the semiconductor are transferred to the counter electrode. A reduction reaction at the counter electrode usually involves an electrocatalyst such as Pt [227]. The electrons combine with the electrolyte ions (usually iodide/triiodide) to complete the circuit [227]. The regenerated electrolyte ions are returned to the dye-sensitized area, where they can repeat the process of accepting electrons from the oxidized chlorophyll molecules [227]. The flow of electrons from the semiconductor to the counter electrode creates an electric current. This current can be collected and harnessed for various applications, such as powering electrical devices or charging batteries.

Dyes extracted from various plant parts are commonly used in scientific studies, with different parts such as fruits, peels, or leaves being utilized for their distinct pigment properties. In documented research, paper [228] details the extraction of dye from the leaves of *Alternanthera dentata*, achieving an efficiency of 0.15%, and from the fruit of *Musa acuminata*, achieving an efficiency of 0.31%. These dyes were utilized as sensitizers for TiO_2_. Additionally, reference [229] discusses the use of papaya leaves as a dye base, attaining an efficiency of 0.07% with ethanol as the solvent.

Expanding on the concept of non-fruit dye sources, paper [230] investigates the use of Hibiscus and Taleng flowers as sensitizers in DSSCs. The study highlights the color properties of the dyes, with Hibiscus flowers producing a red dye and Taleng flowers a blue dye, reporting efficiencies of 0.034% and 0.0183%, respectively.

Further exploring the potential of plant-based dyes, paper [231] reports on the use of barberry fruit and root, as well as the fruit and stem of the carmine herb, without any modification as purifiers or stabilizers. The article, however, does not report the efficiency outcomes of these extracts. On the other hand, paper [232] examines the use of *Curcuma longa* L. as a dye base, with different solvents affecting the efficiency: acetone (0.10%), methanol (0.12%), and ethanol showing the highest efficiency at 0.16%. This research also delved into optimizing parameters like the pH, dye loading period, and dye absorption percentage on the TiO_2_ film.

Moreover, paper [233] details diverse extraction methods such as acid solvent extraction, mechanical extraction, and ultrasound-assisted extraction on Cornelian cherry, presenting a variety of outcomes with the most efficient method using methanol and citric acid achieving 0.98%. Similarly, article [234] describes the use of ethanol as a solvent to extract dyes from *Cassia fistula*, focusing on the flower’s flavonoid pigments, particularly anthocyanins, achieving an efficiency of 0.21%. These studies highlight the nuanced impacts of different extraction techniques and plant parts on the efficiency of plant-based dyes in solar energy applications.

Studies [199,200] employed beetroot as a foundational dye in DSSCs. Their reported efficiencies were relatively low at 0.1788% and 0.49%, respectively. Notably, beetroot was not the primary dye in these investigations, as each study incorporated a secondary dye to enhance performance. Specifically, article [199] detailed the utilization of purple cabbage as an additional dye, achieving an efficiency of 0.38%. Conversely, article [200] used spinach as a secondary dye, combining it with 80% red dye and 20% green dye to achieve a higher efficiency of 0.99%.

Similarly, articles [204,235,236] utilized spinach as the base dye. However, varying outcomes were observed across these studies. Paper [204] reports on using Malabar spinach and red spinach in a dye mixture, with proportions of 20% and 80%, respectively, achieving an efficiency of 0.84%. Article [235], on the other hand, did not combine dyes but examined three separate dyes extracted using acetone as a solvent. This study reported efficiencies of 0.17% for spinach, 0.151% for onion, and 0.104% for purple cabbage. Extending the focus on spinach as a natural dye, paper [236] presented a dye combination that included red spinach and turmeric, reporting efficiencies of 0.134% and 0.378% when used individually. When combined in a 60% turmeric to 40% spinach ratio, the efficiency markedly increased to 1.079%.

Paper [237] explores a natural organic dye derived from the Malaysian *Areca catechu* fruit, assessing the impact of various concentrations of chenodeoxycholic acid (CDCA) and different solvent types on the photovoltaic efficiency of DSSCs. The study reports an initial efficiency of 0.068% with adding 1.5 mM CDCA, which decreases to 0.0118% after 30 min, indicating a decline over time. In contrast, paper [238] focuses on using *Musa acuminata* bracts as a dye base for sensitizers in DSSCs, achieving a higher efficiency of 0.097%. This result is attributed to the effective deposition of ZnO at 3000 rpm and the diffusion of 0.05 mL of dye, demonstrating the varying outcomes dependent on the material and processing conditions used to develop DSSCs.

In another recent study, the flowers of *Mussaenda erythrophylla* were used to extract natural dye in ethanol and deionized water, which was then tested as a photosensitizer. The study also examined the effects of dye concentration and pH on the dye’s optical properties. The optimal photovoltaic performance was achieved using a device fabricated with a P25-TiO_2_ photoanode sensitized by the ethanol extract at a pH of 5. This setup resulted in the highest power conversion efficiency of 0.41%, with a current density of 0.98 mA·cm^−2^, attributed to effective light absorption in the visible spectrum by the Chl a and anthocyanins in the dye [239].

A study focused on extracting natural dyes from Mediterranean olive leaves using a methanol-based solution, examining how different drying times affect DSSC performance. Chlorophyll pigments from Tunisian olive leaves were used as sensitizers. The research showed that longer drying times improve the dye’s ability to transfer electrons to the TiO_2_ semiconductor’s band edge. The dye from the longest drying period (OLDS1) optimally aligned its LUMO with the TiO_2_ conduction band, enhancing electron transfer, reducing charge transfer resistance, and lowering recombination rates compared to shorter drying times. The substrate material, specifically fluorine-doped tin oxide (FTO) and indium-doped tin oxide (ITO), also significantly impacted natural dye-sensitized solar cells’ (NDSSCs’) performance. The FTO/TiO_2_/OLDS1 combination achieved the best results, with a fill factor of 0.73, an efficiency of 0.31%, a short-circuit current density of 0.662 mA/cm^2^, and an open-circuit voltage of 0.642 V. These results highlight the importance of controlled drying times and substrate selection in optimizing NDSSCs with Mediterranean olive leaf dyes [240].

Mahapatra et al., focused on extracting natural dyes from inedible plant sources, specifically the seeds of *Bixa orellana* (kumkum), the pericarp of *Mallotus phillipensis* (kamala) fruits, and ripe fruits of Basella alba (Malabar spinach) [241]. A comparative analysis was conducted to evaluate the efficacy of these extracts as dye sensitizers for solar cells. Key pigments identified in the extracts include bixin in kumkum, rottlerin in kamala, and betalain/betacyanin in Malabar spinach. The performance efficiency of the devices was highest for kumkum at 0.67%, followed by kamala at 0.55% and Malabar spinach at 0.32%. Theoretical calculations, aligned with experimental results, were conducted to assess the thermodynamic parameters like electron injection efficiency and regeneration efficiency using density function theory (DFT). The results indicated that kumkum dye was the most efficient, while Malabar spinach was the least efficient among the tested natural dye sensitizers [241].

### 4.4. Light-Emitting Plants

The global pursuit of robust economic growth has inadvertently fueled environmental issues, including significant energy depletion. As societies demand more energy, the reliance on non-renewable fossil fuels persists. Fortunately, alternative energy sources are being developed to lessen this dependence. These include hydrogen gas, tidal energy, biomass energy, wind energy, geothermal power, biofuels, and solar power [242]. One innovative solution that could illuminate areas without electricity is the development of light-emitting plants.

The concept of light-emitting plants has captivated the scientific community’s interest since 1986 as a promising alternative energy source [243]. The development of light-emitting plants has primarily involved genetic modification. This process includes the integration of genes from bioluminescent organisms such as fireflies (*Photinus pyralis*) [243], luminous bacteria (*Photobacterium leiognathi*) [244], and bioluminescent fungi (*Neonothopanus nambi*) [245]. These genes enable the plants to produce light naturally, mimicking the luminescence mechanisms found in these organisms.

Beyond the genetically modified plants, researchers have also utilized strontium aluminate (SrAl_2_O_4_) NPs to develop light-emitting plants.

SrAl_2_O_4_ or phosphor, a widely studied photoluminescent compound, has garnered significant interest due to its exceptional afterglow intensity, prolonged duration of luminescence, and superior persistence times relative to other phosphors. Additionally, it exhibits favorable chemical and physical properties, including high stability, excellent quantum yield, chemical and biological inertness, and the capability of indefinite recharging by both ultraviolet and visible light sources. The application of SrAl_2_O_4_ in developing light-emitting plants, focusing on optimizing its use and assessing phytotoxic effects was investigated. SrAl_2_O_4_, known for its long-lasting glow and easy rechargeability by visible light, was tested for its efficacy and impact on plant health. The process begins with the preparation of a suspension of strontium aluminate particles. These particles are then injected into the plant tissues, often into the vascular system of the plants. The injection can be facilitated using a suitable carrier medium, ensuring that the particles are evenly distributed throughout the plant. Once injected, the SrAl_2_O_4_ particles are taken up by the plant’s vascular system, similar to how nutrients and water are transported. The particles travel through the xylem and phloem, dispersing throughout the plant’s tissues, including leaves, stems, and flowers [243,246].

When the SrAl_2_O_4_ particles within the plant tissues are exposed to a light source (natural sunlight or artificial light), they absorb the light energy and become excited. The excited electrons in the strontium aluminate then gradually return to their ground state, releasing the stored energy as visible light. This process is known as photoluminescence, specifically phosphorescence, due to the prolonged emission of light even after the excitation source is removed [243,246].

The duration and intensity of the light emission depend on the properties of the strontium aluminate particles, such as their size, concentration, and the specific dopants used to enhance their luminescence. Typically, SrAl_2_O_4_ doped with europium and dysprosium exhibits a bright green afterglow that can last for several hours [243,246].

By integrating these NPs into five diverse plant species, they achieved a remarkable photoemission rate of up to 4.8 × 10^13^ photons per second. Consequently, the use of SrAl_2_O_4_ NPs emerges as a promising and innovative method for advancing light-emitting plants technology, offering an alternative pathway for enhancing bioluminescence in plants [246]. This approach not only diversifies the techniques used in the development of light-emitting plants but also expands the potential applications of bioluminescent technology in environmental and aesthetic contexts.

The application of SrAl_2_O_4_ in developing light-emitting plants, focusing on optimizing its use and assessing phytotoxic effects, was investigated. SrAl_2_O_4_, known for its long-lasting glow and easy rechargeability by visible light, was tested for its efficacy and impact on plant health. The authors used different SrAl_2_O_4_ products: product 1 (39.6% SrO, 37.6% Al_2_O_3_, 20.03% P_2_O_5_, 0.151% CaO, 0.173% SiO_2_, 0.921% Eu_2_O_3_, 1.53% Dy_2_O_3_), product 2 (33.6% SrO, 60.1% Al_2_O_3_, 3.09% P_2_O_5_, 0.0447% CaO, 0.540% SiO_2_, 1.61% Eu_2_O_3_, 1.02% Dy_2_O_3_), and product 3 (47.2% SrO, 43.9% Al_2_O_3_, 4.13% ZrO_2_, 0.0447% CaO, 0.649% SiO_2_, 1.80% Eu_2_O_3_, 2.28% Dy_2_O_3_). In the experiments, 3 mL of 5% (*w*/*v*) SrAl_2_O_4_ solutions were injected into the stem of *Ipomoea aquatica*. Products 2 and 3 of SrAl_2_O_4_ induced oxidative stress in the plants, while product 1 elicited a defensive response by increasing levels of pipecolic acid and salicylic acid. Conversely, the plants treated with products 2 and 3 displayed a reduction in salicylic acid by approximately 0.005- and 0.061-fold, respectively, and an accumulation of malondialdehyde (MDA) between 10.00 and 12.00 μmol g^−1^ fresh weight, compared to control plants. Furthermore, it was determined that a 15% concentration of SrAl_2_O_4_ could sustain enhanced photoemission up to 18 times the baseline for 50 min. Among the SrAl_2_O_4_ formulations, product 1 shows promise as a viable material for the further development of light-emitting plants, offering a potentially effective and environmentally friendly lighting alternative [243].

### 4.5. Engineered/Bionic Plants for Detection of Explosives and Pollutants

Engineered plants designed for detecting pollutants and explosives utilize a combination of genetic modification, biosensing technologies, and interfacing with engineered nanomaterials. This innovative approach begins with the identification of specific targets such as heavy metals, volatile organic compounds, pesticides, and explosive compounds [247]. Genetic modification plays a critical role in developing these plant sensors. Researchers select genes from various organisms, including bacteria, other plants, or synthetic constructs, that respond to the presence of target pollutants or explosives [248]. These genes typically encode proteins or enzymes whose expression or activity changes upon exposure to contaminants [249,250]. Specific promoter regions, which activate in response to these substances, control the expression of the reporter genes, ensuring that the plant’s response is specific to the presence of the pollutants or explosives [251,252,253]. Reporter genes, such as those encoding fluorescent proteins (e.g., GFP—Green Fluorescent Protein), luminescent proteins (e.g., luciferase), or colorimetric enzymes (e.g., β-glucuronidase), are introduced into the plant genome [251,252,253]. The expression of these genes leads to visible changes in the plant, such as fluorescence, luminescence, or color change, indicating the detection of pollutants or explosives [248]. The genetic constructs are introduced into the plant genome using techniques like Agrobacterium-mediated transformation and biolistic transformation (gene gun). *Agrobacterium tumefaciens*, a bacterium that naturally transfers DNA to plants, is commonly used for dicotyledonous plants. In contrast, the gene gun method is suitable for monocotyledonous plants and other species less susceptible to Agrobacterium-mediated transformation [254,255]. Following the introduction of genetic constructs, genetically transformed cells are regenerated into whole plants through tissue culture techniques [254,255]. These plants are then selected and propagated to ensure the stable integration and expression of the introduced genes.

In addition to genetic modification, interfacing plants with nanomaterials enhances their detection capabilities [247]. Nanomaterials such as single-walled carbon nanotubes (SWCNTs), quantum dots, and metal NPs exhibit unique optical or electronic properties in the presence of specific pollutants or explosives [256]. These nanomaterials are functionalized with specific molecules or polymers that interact with the target substances, increasing their sensitivity and specificity [257]. The functionalized nanomaterials are integrated with plant tissues, creating hybrid biosensors that amplify detection signals and improve the accuracy and sensitivity of the plant sensors [258]. The detection mechanism in these engineered plants involves the expression of specific proteins that interact with the target pollutants or explosives [259]. These interactions trigger a cascade of biochemical events and a response from the nanomaterials, leading to the activation of reporter genes [256]. The combined response from the plant proteins and nanomaterials results in enhanced detectable signals such as fluorescence, luminescence, or color change [258]. The intensity or presence of these signals correlates with the concentration of the pollutants or explosives, providing a measurable indication of contamination [260].

Most plant sensors fabricated through plant nanobionics utilize SWCNTs and the associated corona phase molecular recognition (CoPhMoRe) technique for target detection [261,262].

SWCNTs are fluorescent nanomaterials that emit in the near-infrared region and exhibit superior photostability compared to traditional fluorescent dyes. The fluorescence of SWCNTs occurs near or within the second near-infrared window (NIR-II, 1000–1700 nm), which reduces interference from chlorophyll autofluorescence and allows for high-resolution plant bioimaging [263,264]. Due to their ultralow photobleaching, SWCNTs are ideal for the long-term monitoring of plants [265]. The CoPhMoRe technique relies on the interactions between the polymer wrapped around the NPs and the target molecule, which induces a shift in the fluorescence of the nanoparticles [266].

Plants can be engineered to detect explosives and pollutant agents [267]. Wong et al., described the development of a nanobionic plant based on living spinach (*Spinacia oleracea*) and a pair of NIR fluorescent nanosensors—SWCNTs conjugated to the peptide Bombolitin II to recognize nitroaromatics *via* infrared fluorescent emission, and polyvinyl-alcohol (PVA) functionalized SWCNTs acting as an invariant reference signal—embedded within the plant leaf mesophyll [268]. This bionic spinach can detect nitroaromatic compounds in ambient groundwater and can send information to a smartphone by infrared communication. The nitroaromatic compounds are relatively rare in nature and have been introduced into the environment (soil and groundwater) especially by human activities, and they are toxic, mutagenic, and can be easily reduced into carcinogenic aromatic amines [269]. As known, some dyes (such as Picric acid (1,3,5-trinitrophenol), a yellow dye for fabrics), pesticides, and explosives (such as TNT (2,4,6-trinitrotoluene), a major component of many composite explosives) contain nitroaromatic compounds [269].

Furthermore, photoluminescent metal–organic frameworks (MOFs) were cultivated within a living *Syngonium podophyllum* plant by submerging its roots in a solution containing disodium terephthalate and terbium chloride hexahydrate over a continuous period of 12 h [270]. This process did not harm the plant’s survival. Researchers then used app-assisted live MOF–plant nanobiohybrids to detect and measure harmful metal ions and organic contaminants. The study showed that the plants acted as self-powered preconcentrators, using their fluid transport systems to gather pollutants around the MOFs, altering fluorescence intensity. The nanobiohybrids displayed high selectivity and sensitivity (0.05–0.5 μM) for Ag^+^, Cd^2+^, and aniline, increasing fluorescence. For Fe^3+^ and Cu^2+^, fluorescence decreased in the 0.05–10 μM range. Measurement precision was 5% and accuracy was 10%. The software converted plant luminescent signals into digital data on a smartphone, enabling the real-time detection of environmental toxins with high accuracy and sensitivity. These results suggest that combining synthetic and living materials can create advanced sensors for on-site environmental pollutant detection [270].

Engineered plants for pollutants’ and explosives’ detection offer several applications and benefits. In environmental monitoring, these plants provide the real-time detection of contaminants, allowing for rapid assessment and remediation of polluted sites. In security applications, plants engineered to detect explosives can enhance safety by identifying hazardous compounds in public spaces and conflict zones. This approach is cost-effective and sustainable, leveraging the natural sensing capabilities of plants to continuously monitor large areas without the need for frequent sampling or complex instrumentation. The integration of nanomaterials significantly enhances the sensitivity and specificity of detection. These plants can also serve as educational tools, raising awareness about environmental and security issues, and as research tools, aiding in the study of plant responses to environmental stressors.

## 5. Plants in the Agricultural Sector

Plants engage in uninterrupted gas and fluid exchange with the environment *via* their leaves and roots [246]. The dynamic oscillations in the environment closely correlate with the growing state of plants [271], and plants can also indicate the variations in their surroundings. Conventional methods for studying plant characteristics include “3S” technologies, which consist of remote sensing (RS), spatial information systems, and global positioning systems [272]. Other techniques include spectroscopy, machine vision, and digital picture capture/analysis [273]. These technologies are appropriate for detecting and measuring plant characteristics at a large scale, such as chlorophyll and nitrogen levels, canopy information, leaf area index, and pest infestation [274]. Nevertheless, these technologies are unable to acquire crucial data about plant communication, reactions to environmental pressures, and internal physiological signal alterations at the micro-nano level [275].

Some plants can survive extreme dehydration and quickly recover with water, providing insights into water management in crops. Resurrection plants, such as those from the genus Selaginella, can lose almost all their water content during drought and then rapidly rehydrate when water becomes available [276]. Researchers are studying these plants to understand the molecular and physiological mechanisms that enable such resilience. This knowledge can be applied in developing drought-resistant crops through genetic engineering or breeding programs, improving water use efficiency in agriculture. Leguminous plants, such as peas, beans, and clover, form symbiotic associations with *Rhizobium* bacteria in their root nodules [277]. These bacteria convert atmospheric nitrogen into forms that are usable by plants, reducing the need for synthetic fertilizers. Biomimetic applications include developing crops with enhanced nitrogen-fixing capabilities or creating biofertilizers that mimic this natural process, promoting sustainable agriculture [278]. Plants have evolved complex immune systems, including pattern recognition receptors (PRRs) that detect pathogen-associated molecular patterns (PAMPs) and trigger defense responses [279]. Understanding these mechanisms can lead to the development of crops with improved disease resistance through genetic modification.

Additionally, biomimetic approaches can inform the design of novel pest management strategies that enhance the plant’s innate immune responses. Root architecture plays a critical role in nutrient and water uptake, as well as plant stability. Researchers are using biomimicry to design root systems that maximize efficiency. For example, understanding the root branching patterns and growth dynamics of certain plants can inform breeding programs aimed at developing crops with more effective root systems [280]. This can lead to enhanced nutrient uptake, reduced need for fertilizers, and better resilience to environmental stresses [281]. C4 plants [282], such as maize and sugarcane, and CAM plants [283], like cacti and succulents, have evolved efficient photosynthetic pathways to thrive in hot and arid environments. By studying these pathways, scientists aim to develop crops that can perform photosynthesis more efficiently under suboptimal conditions. Genetic engineering or selective breeding to incorporate traits from C4 or CAM plants into staple crops could significantly enhance agricultural productivity and sustainability.

Before the twentieth century, crops were safeguarded against pests and hungry animals using traditional methods, rather than the synthetic chemicals known as pesticides that were later introduced [284]. The extensive utilization of synthetic organic chemicals as pesticides in recent decades has resulted in the pervasive contamination of all environmental and biological components with minute quantities of small organic compounds [285]. The cumulative impact of these compounds on human health and the overall stability of intricate ecological systems remains largely unknown. It is argued that the existence of small quantities of these substances in human food could potentially harm the well-being of susceptible individuals, particularly during the developmental and early phases of life [286]. However, there is no unanimous agreement on this issue. The demand from consumers, primarily in developed Western nations, for food produced under perceived healthier conditions than mass-production has led to a resurgence of interest in traditional, lower-yield methods like “organic farming”. This has created a need to safeguard consumers from unsupported claims about food quality [286]. Producers are now issuing voluntary codes, and international organizations provide guidelines such as Codex Alimentarius. Another catalyst for the revival of “traditional” farming methods is the recognition that they can be effectively combined with “modern” techniques to achieve higher yields while minimizing environmental impact, reducing the use of costly and selective Plant Protection Products and decreasing food contamination from residues [287]. One reason to look for alternatives to traditional pest control methods is the recognition that future generations of pests may become resistant to current pesticides due to an ongoing battle between human scientific understanding and the natural evolution of organisms. Given that even naturally occurring substances used to combat plant and food parasites are inherently toxic to the intended species and several others, including humans, it is essential to conduct risk assessments before utilizing them. This ensures that their potency can be fully harnessed without posing unnecessary risks to producers, consumers, and the global environment [288,289]. It is crucial to thoroughly comprehend the extent and constraints of utilizing both natural and manufactured organisms together with their hazardous substances as pesticides, as this has been a recent trend.

The initial significant synthetic organic pesticide was Dichlorodiphenyltrichloroethane (DDT), which was originally created by German chemist Ziedler in 1873 [290]. The insecticidal property of the substance was first identified by Paul Muller, a chemist from Switzerland, in 1939. During its initial stages, DDT was widely praised for its remarkable properties such as its ability to target a wide range of organisms, its long-lasting effects, its inability to dissolve in water, its affordability, and its ease of use [291].

While the ban on using DDT in agriculture was suggested by the Stockholm Convention on Persistent Organic Pollutants [292], its production and use were still allowed to control disease-carrying organisms, following the strict recommendations and guidelines of the World Health Organization (WHO). However, this was only permitted when there were no other locally available options that were safe, effective, and affordable [293]. DDT is still employed in many underdeveloped countries to combat malaria and other tropical diseases. It is applied by spraying the interior walls to eliminate disease-carrying organisms [294].

But classical pesticides are very harmful to humans and to the environment, so “green” alternatives have been developed. An eco-friendly strategy is using plant extracts and essential oils as biopesticides (e.g., fungicides, herbicides, repellents, insecticides, nematicides, etc.).

The plant-derived pesticides have many advantages compared to synthetic ones: low cost, biodegradability, and eco-friendliness. They are less hazardous to humans and the environment, being a green alternative to synthetic pesticides. The plants contain many bioactives (e.g., polyphenols, flavonoids, terpenes, alkaloids, cyanogenic glucosides, quinones, saccharides, amides, aldehydes, thiophenes, amino acids, polyketides, etc.) which confer them pesticidal properties [295].

Some essential oils originating from cedarwood, eucalyptus, cinnamon, cloves, garlic, geranium, lemongrass, citronella, peppermint, rosemary, sesame, and thyme were used as biopesticides [296].

Aquatic plants such as duckweed (*Lemna minor*), water hyacinth (*Eichhornia crassipes*), muskgrass (*Chara* spp.), hydrilla (*Hydrilla verticillata*), and water lettuce (*Pistia stratiotes*) are also used as biopesticides since they produce allelopathic chemicals that have highly potent inhibitory properties against pathogens. The extract of the aquatic plant Neem (*Azadirachta indica*) kills many insects [297].

The medicinal and aromatic plant peppermint (*Mentha piperita*) is an attractive source of biopesticides. Peppermint-based nanobiopesticides with enhanced stability, solubility, and pesticidal potential were prepared by Jahan et al. [298]. The *M. piperita* nanosuspensions were obtained by the antisolvent precipitation method. *M. piperita* nanosuspensions stabilized with sodium lauryl sulfate showed good antibacterial activities against two phytopathogenic bacterial strains, *Clavibacter michiganensis* and *Pseudomonas syringae*, high antifungal action against *A. niger* and *F. oxysporum*, and pesticidal activity against the stored-grain insects *Tribolium castaneum* and *Sitophilus oryzae*.

Other researchers prepared Ag NPs [299] and ZnO NPs [300] by using *Mentha piperita* extract. These NPs exhibited greater insecticidal and antimicrobial potential than mint extract.

Plant nanobionics represents a pioneering field in agricultural technology where nanotechnology is integrated with plant biology to create enhanced or entirely new capabilities within plant systems. This interdisciplinary approach leverages the natural biological processes of plants and augments them with the unique properties of nanomaterials, aiming to revolutionize agricultural practices, improve crop resilience, and increase productivity. Nanobionics involves the integration of engineered nanomaterials into plant cells and organelles to modify or enhance their overall functions [301]. Consequently, the field of nanobionics has developed to include a range of plant-based applications, such as the utilization of plants as effective delivery systems for nutrients and hormones, the development of plant-based nanosensors, and the creation of self-illuminating plants [302].

The integration of nanobionics into plant systems for agricultural applications has been shown to enhance various plant parameters such as seed germination, germination time, root growth, biomass, leaf number, and fruit production [303]. These enhancements lead to increased yields of plant-derived polymeric materials, including polysaccharides. These biopolymers are characterized by their specific structural properties, reduced financial costs, non-toxic nature, ease of modification, biocompatibility, and widespread availability, making them highly valuable in industries such as food processing, pharmaceuticals, and nanomedicine [304,305]. Furthermore, various types of nanomaterials, including metals, metal oxides, carbon, and polymer-based nanoparticles, have been extensively used to improve the development, growth, and crop yields [305]. Nano-based compounds offer significant advantages for agricultural productivity through their capacity to improve nutrient efficiency in crop development. These materials, whether used alone or in combination with traditional pesticides or fertilizers, deliver nutrients more effectively. Nano-sized materials act as adsorbents that release nutrients gradually and efficiently [306]. Recent studies, such as the use of graphene oxide-proline (GO–Pro) nanoparticles, have demonstrated their ability to enhance the performance of Moldavian balm and its essential oil production under increased salinity conditions, illustrating a novel method to enhance crop stress tolerance [307].

Further research has shown that diverse applications of NPs can lead to significant agricultural benefits. For example, exposure of *Cyamopsis tetragonoloba* to zinc oxide nanoparticles at 10 mg/L for 4–6 weeks resulted in increased biomass accumulation and improved nutrient uptake and growth physiology [308]. Similarly, foliar application of nano-compounds like Zn, Fe, and NPK combinations at 20 L/plot significantly boosts biomass and seed yield over 30 days [309]. Copper oxide (CuO) NPs at 150–340 µg/mL concentrations have been found to promote shoot and root development in *Beta vulgaris* L. effectively [310]. Additionally, foliar applications of a mixed combination of ZnO NPs and Fe_3_O_4_ NPs in various dosages (30, 60, and 90 mg/L) over three days have been shown to reduce salinity levels and significantly improve plant growth. For instance, foliar application of zinc nanoparticles on *Punica granatum* at 120 mg Zn L^−1^ markedly increased fruit yield [311]. Nitrogen, phosphorus, and potassium (NPK)-based nanoparticles also significantly enhance growth in *Triticum aestivum*, affecting shoot and root development as well as fruit production [312]. Furthermore, an NPK-based NP formulation applied foliarly at 500 ppm for 21 days significantly increased the total saccharide content in wheat grains [313].

*Coffea arabica* exposed to a ZnO NP foliar spray at 15 mg/L for 40–45 days showed increased photosynthesis and biomass production [313]. In addition, ZnO supplementation combined with phosphorus not only boosted biomass, protein, and pigments for photosynthesis but also provided protection against oxidative stress [314]. Additionally, silica-based NPs have been utilized to transmit genes and their inducing chemicals into tobacco leaves and protoplasts, showcasing another innovative application of nanotechnology in agriculture [315].

Enhancing photosynthesis through plant nanobionics represents a groundbreaking approach to agricultural and environmental challenges, aiming to boost crop productivity and carbon fixation efficiency beyond natural limits. The capacity of NPs to penetrate plant cells and enhance photosynthetic efficiency is influenced by their size and composition [246]. Carbon-based nanomaterials, in particular, have been noted to impact various plant functions [316]. These NPs offer protective benefits, such as shielding plants from UV radiation by reducing UV absorption, as evidenced in multiple studies [316,317,318]. For example, exposure to carbon NPs at concentrations of 40–60 mg/L has been shown to promote plant regeneration and improve the root/bud ratio in *V. zizanioides* [303]. Conversely, carbon-based nanodots used on *Zea mays* resulted in decreased root mass and smaller shoots but enhanced biochemical synthesis and antioxidant activities [319]. Similarly, roots of *S. lycopersicum* treated with graphene were found to be longer than those of control plants [320,321], and while root elongation in *Triticum aestivum* was significantly enhanced, the growth of root hairs was inhibited when exposed to graphene [322,323].

Nanotechnology, a prominent area of research globally, holds significant promise in facilitating plant–electronic device communication and activation. This technology can enhance people’s understanding of a plant’s physiological condition and the evolving dynamics of its environment [324]. In the context of contemporary agricultural progress, nanosensors have successfully accomplished precise, instantaneous, and highly detailed monitoring of individual plants at a microscopic level [324]. These nanosensors convert the chemical signals produced by plants into optical, wireless, or electrical signals, enabling people to exert improved control over all aspects of agricultural production.

Plants persist in an ever-changing environment, which might occasionally be unfavorable for their development [325]. The humidity level varies in response to variations in the surrounding temperature, and the opening and closing of leaf stomata during photosynthesis is directly influenced by changes in temperature and humidity. Additionally, plants are susceptible to the detrimental effects of pests and diseases throughout their development [326]. Overall, these variable environmental conditions will induce a range of cell responses, ultimately impacting plants’ physiological processes. Plants frequently display distinctive and intricate reactions to changes in their surroundings, employing intercellular communication to combine signals from many tissues and organs [327]. Typically, these signals involve the regulated release of plant hormones and the quick propagation of cell membrane polarization waves. Monitoring these signals can offer insights into changing environmental conditions [328,329]. Hence, employing nanosensors for plant communication proves to be beneficial in comprehending their growth mechanisms, while the real-time monitoring of these signals is immensely important in enhancing plant productivity.

Among all the environmental elements, water is crucial for plant growth due to its regulation of the photosynthetic and transpiration processes [330]. Compared to sensors placed in the soil or among plants, wearable nanosensors that are directly attached to plants can more accurately and immediately detect slight changes in the water levels of the plants themselves, particularly in the early stages. Based on this, academics have conducted multiple studies. Oren et al., employed graphene-based nanosensors, which were applied to the rear surfaces of maize plant leaves, to detect variations in relative humidity (RH) on the leaf surface [331]. When the leaves are irrigated, the relative humidity (RH) changes, and this shift is directly reflected in the resistance of the nanosensor.

Living plants need good-quality water to grow and survive. Thus, the purification of waste waters and their reuse in irrigation are of great ecological and economic importance.

## 6. Phyto-Based Approaches in Wastewater Treatments

Plants have long been utilized for their natural purification properties, with various parts of plants being used to remove contaminants from wastewater. This section will provide an in-depth analysis of the functionality of different plant products used in wastewater treatment, explaining how they work and their effectiveness.

For example, *Moringa oleifera* seeds contain proteins that act as natural coagulants. When the seed powder is added to water, the positively charged proteins bind with negatively charged particles, such as clay, silt, and bacteria, causing them to clump together (flocculate) and settle out of the water. Moringa seeds have been shown to effectively reduce turbidity, bacterial content, and organic matter in water. This makes them an eco-friendly and cost-effective alternative to chemical coagulants like aluminum [332].

Activated carbon derived from coconut shells has a high surface area and porosity, making it effective at absorbing a wide range of contaminants. This includes heavy metals, organic pollutants, and chlorinated compounds. Coconut shell activated carbon is particularly effective in removing color, odor, and taste from water, as well as reducing levels of heavy metals such as lead and mercury [333].

Banana peels contain functional groups such as carboxyl, hydroxyl, and amino groups, which can bind to heavy metals. The peels can be processed into a bio-sorbent material that adsorbs metals from wastewater. Studies have demonstrated that banana peels can effectively remove heavy metals like lead, copper, and cadmium from water. This provides a low-cost and biodegradable solution for treating industrial wastewater [334].

The mucilage from cactus plants, particularly *Opuntia* spp. (Cactus), acts as a natural flocculant. It can bind to suspended particles and heavy metals, facilitating their removal through sedimentation. Cactus mucilage has been effective in reducing turbidity, heavy metal concentrations, and pathogens in water. Its use is especially advantageous in arid regions where cacti are abundant and water resources are scarce [335].

Water hyacinth (*Eichhornia crassipes*) plants absorb and accumulate pollutants from water through their root systems. They are particularly effective in uptaking heavy metals, nutrients, and organic compounds. Water hyacinth has been widely used in constructed wetlands and natural water bodies to treat wastewater. It can significantly reduce levels of nitrogen, phosphorus, heavy metals, and organic pollutants, though care must be taken to manage its invasive growth potential [336].

Many publications from diverse countries, such as Brazil, Egypt, Malaysia, Portugal, India, Romania, China, and Singapore, were examined to explore the possible use of aquatic plants for this specific purpose [337]. This study of Anand et al., employed a systematic mapping review strategy in the given setting. The primary objective of this review paper is to examine the latest advancements in wastewater phytoremediation technologies, identify their limitations, and pinpoint areas within the phytoremediation process that may necessitate future investigation. Until this point, over 30 aquatic plants have been mentioned in this article. This report offers sufficient information to assist researchers in selecting suitable plants and determining the optimal hydraulic retention duration for experiments on the phytoremediation of wastewater.

Wastewater treatment has become essential in response to the growing need for potable water and water for industrial use. Complexation, precipitation, ion exchange, advanced oxidation processes (AOPs), membrane filtration, reverse osmosis, activated carbon adsorption, ultraviolet (UV) photolysis/photocatalysis, and electrodialysis are all conventional technologies used for wastewater treatment. Efforts are being made to enhance existing wastewater treatment systems and create new solutions that are cost-effective and energy-efficient, to deliver potable water in ecologically friendly ways through wastewater treatment and desalination [338,339]. Protein nanofibrils, covalent organic frameworks (COFs), metal–organic frameworks (MOFs), graphene, carbon nanotubes, fluorous oligoamide nanorings, and artificial water channels (AWCs) are among the sophisticated materials employed in wastewater treatment [340,341,342]. Nevertheless, these materials incur substantial manufacturing expenses and involve a complex synthesis procedure.

There has been a lot of recent attention on biopolymers since they are found in large quantities in the environment and have the potential to be used as a source for producing nanofibers that are based on biological materials. Hydrophilicity, mechanical and chemical stability, and chemical modifiability are notable characteristics of these substances, which prevent fouling. Extensive study has focused on the possible application of biopolymers in water purification to remove different types of contaminants [343]. Plant-derived biopolymers, including cellulose and hemicellulose, have been thoroughly investigated for this particular use [344]. Scientists have prioritized cellulose as a biopolymer for water purification, including membrane filtration, micropollutant elimination, dye decomposition, and oil–water separation. This is due to their ability to adsorb substances, compatibility with living organisms, disinfection properties, and absence of harmful effects [345,346,347,348].

Compared to typical water treatment materials including activated carbon, clays, metal sulfides, carbon-based nanomaterials, zeolites, and mesoporous silica, the phyto-based materials (PBMs) provide multiple advantages [349,350]. PBMs represent a viable “green” alternative for wastewater treatment. Here are several significant benefits of PBMs: (i) they are naturally abundant, (ii) they have the ability to eliminate heavy metals and other small amounts of impurities, (iii) they readily break down through biological processes, (iv) they are inexpensive, and they demonstrate high effectiveness in terms of heat generation and power usage [349]. Due to their inherent qualities, they are highly ideal for use as adsorbents and materials for water filtration [351]. Although bio-based products offer numerous benefits, several challenges remain. The inefficiency and suboptimal utilization of inexpensive, readily available feedstocks such as lignocellulosic materials impede the reduction in production costs for PBMs to a feasible level for large-scale production. Appropriate techniques for creating and modifying PBMs to tailor their properties to desired chemicals have not yet been discovered. Key considerations when studying PBMs include pollutant characteristics, removal speed and processes, environmentally friendly synthesis methods, efficient utilization, and the recyclability of biomaterials [352,353,354].

There are potential green strategies for removing dyes, specifically based on phyto-derived (nano)materials like cellulose, and other plant-derived nanofibers. Conventional methods such as adsorption, membrane filtration, coagulation, chemical precipitation, flocculation, and ion exchange can remove dyes. Various methods can be used to synthesize natural nanofibers, and one of these methods is electrospinning. Electrospinning has several advantages, including efficiency, cost-effectiveness, and great reproducibility. Furthermore, chemical alteration is another feasible approach [355]. This work extensively discusses the improvement in characteristics achieved by modifying plant-based materials with different groupings and examines various bio-based materials and bio-based nanofibers used for water purification.

The phytosynthesized semiconductor metal oxide (MO) NPs such as ZnO NPs, TiO_2_ NPs, CeO_2_ NPs, and SiO_2_ NPs possess high photocatalytic activity, and they have been used in wastewater purification through a photocatalytic contaminant degradation approach. The green CuO NPs synthesized using an extract of *Moringa stenopetala* seeds were reported to photodegrade congo red and alizarin highly, and the CuO NPs biosynthesized from *Amaranthus dubius* leaf extract photodegraded methylene blue [21]. CuO NPs synthesized with the leaf extract of *Eucalyptus globulus* proved to be good adsorbents of methyl orange from aqueous media [356].

## 7. Botanical Robots

Botanical robots represent a cutting-edge fusion of living plant components with mechanical or electronic elements, creating innovative devices that harness the unique capabilities of plants. These biohybrid systems leverage plants’ natural abilities to sense and respond to environmental stimuli, self-heal, and grow, enabling a wide range of applications. Botanical robots are designed by integrating living plants or plant tissues into robotic frameworks. This integration can vary from utilizing plant materials for structural parts to developing sophisticated biohybrid systems where living plants actively interact with robotic mechanisms. The primary goal is to combine the best features of both biological and mechanical systems to create functional and sustainable devices.

Biodegradable materials decompose and break down in the natural environment as a result of the activity of soil microorganisms. Therefore, the robot or actuator must establish physical touch with the soil or be submerged within it. On the other hand, if it lands on a stone or in sand, or becomes trapped on a tree, the deterioration process will be postponed. There is a possibility that the substance can undergo decomposition in the environment at normal room temperature. Nevertheless, the robots and actuators may become non-functional in such instances before accomplishing the task.

The “robots” created from plants can actively regulate the initiation of biodegradation, rather than passively undergoing natural progressive degradation. This study specifically examined the processes of germination and predation [357]. A biodegradable robot utilizes edible actuators as a source of nourishment for animals and plants. While the robot is in operation, the food component is concealed beneath a coating layer. Upon achieving the target, the covering dissipates, revealing the food component. This approach enhances the process of biodegradation by subjecting the robot to predation [357]. The details of this strategy will be elaborated upon in a subsequent correspondence. Another approach, influenced by the germination process of *Ophiocordyceps*, is inducing the soft actuators to initiate germination towards the end of their life cycle [358,359]. Ophiocordyceps is a fungal genus that parasitizes insects, using their bodies as hosts and manipulating their behavior for reproduction and migration. This inspired the development of germinating robots. The study uses a soft actuator with germinated seeds that integrate into nature once robot operations end. Predators may move these seeds, aiding in natural decomposition. During growth, the germinated seeds embed in the soft actuator, accelerating its degradation. Germination is regulated by exposing the internal environment to the external environment through a biodegradable bag in the soft actuator, triggered by rainwater [359].

To make decisions that balance protecting the environment with prioritizing their own well-being, plants collect and analyze information about their surroundings. These choices are communicated by electrical signals that travel within and between cells [327]. These messages mostly take the form of action and variation potentials in response to various stimuli, such as humidity, temperature, light intensity, and mechanical vibrations. While it is widely known that certain plants, like the *Mimosa pudica*, can respond electrically to brief environmental stimuli (like touch), nothing is known about how these plants would respond electrically over the long term to gradual environmental changes [327]. Here, over the course of around five days, a multi-source monitoring system has been constructed to gather and store electrical signals from the plant *Mimosa pudica* as well as the temperature and humidity of the surrounding environment. The environmental temperature and variation potential (VP) from *Mimosa pudica* are displayed on a real-time dashboard. The VP replicates, with a corresponding delay, variations in the ambient temperature. The extended physiological studies imply that the plant *Mimosa pudica’s* ability to sense external temperature may be observed, and that it is most likely powered by bioelectricity.

Bio-inspired robotics occasionally replicates the growth-based movement of plants; however, growth is just one aspect of a more comprehensive system of movement guidance and control. We contend that understanding “information” and “control” in ecological psychology may effectively explain how a plant navigates its surroundings and offer a framework for designing ecological robotics inspired by plants. Within this endeavor, we shall delineate several control laws and place particular emphasis on the category of control laws recognized by tau theory, such as time to touch [360].

## 8. Concluding Remarks and Future Directions

This paper presented some interesting and unusual aspects related to the Plant Kingdom and its impact on our lives, seen from a “green” perspective. One of the recent scientific trends is the development of bionic plants for various applications such as botanical robotics, light-emitting plants, and sensing applications like the detection of explosives or toxic compounds.

The distinctive spectral characteristics of vegetation are critical for various applications, including remote sensing and ecological monitoring. These characteristics enable the effective differentiation of vegetative cover from non-living and man-made surfaces, which is vital for designing effective camouflage patterns in both civilian and military contexts. The advancement of detection technologies, particularly hyperspectral imaging, challenges the effectiveness of traditional camouflage, pushing for the development of materials that visually match and spectrally replicate the surroundings. This involves mimicking the complete spectral range from visible to near-infrared, enhancing camouflage against modern detection methods. Furthermore, the green hue of leaves, primarily resulting from chlorophyll, poses additional challenges for artificial replication due to its unique light absorption and reflection properties. Overall, the complexity of natural vegetation’s spectral properties necessitates sophisticated approaches in developing biomimetic materials for effective spectral simulation and camouflage.

Moreover, vegetal scaffolds are used in the biomedical field to biofabricate artificial organs, being a viable and green alternative to transplants. Scaffolds play a pivotal role in tissue regeneration, and, in this review, we discussed those derived from plant materials through the decellularization process. Decellularized scaffolds offer enhanced biocompatibility and biodegradability compared to synthetic composite scaffolds. Additionally, they are more cost-effective and generate fewer waste products. Unlike composite scaffolds, which require complex machinery, decellularized scaffolds can be produced with basic equipment such as a shaker incubator for the decellularization process and a lyophilizer for preservation. Given these advantages, plant-based decellularized scaffolds hold significant potential for future developments in tissue regeneration and other biomedical applications.

While DSSCs using natural dyes present notable benefits, they have struggled to penetrate the solar market due to their lower efficiency compared to traditional silicon solar panels. This article conducts a comprehensive literature review to gain broader insights into DSSCs and identify critical issues related to dye selection and synthesis methods. Future directions for developing DSSCs using natural dyes can focus on several key areas to enhance their market viability and overall efficiency. Research should aim to improve the chemical stability of natural dyes under solar exposure, increasing their longevity and performance consistency. Innovations might include the development of new dye formulations or encapsulation techniques that protect dyes from degradation due to environmental factors. Further studies are needed to optimize natural dyes’ extraction and purification processes to maximize their photovoltaic efficiency. This includes refining solvent choices, extraction times, and temperatures to enhance the yield and quality of the active components. Exploring co-sensitization strategies that combine multiple natural dyes or a mix of natural and synthetic dyes could lead to broader absorption spectra and higher efficiencies. This approach may also help in overcoming the limitations of single-dye sensitization. Developing nanostructured photoanodes with tailored surface properties can enhance dye loading and light-harvesting efficiency. Research into nano-engineering the TiO_2_ surface or exploring alternative materials could provide significant gains in performance. Investigating hybrid systems that combine the benefits of natural dyes with other photovoltaic technologies, such as perovskite solar cells, could lead to breakthroughs in efficiency and stability, making DSSCs more competitive. Comprehensive studies assessing the environmental impact and economic viability of natural dye-based DSSCs are crucial. This includes lifecycle analyses and cost–benefit studies compared to conventional photovoltaic technologies. Innovations in the overall design and assembly of DSSCs, including better methods for sealing and encapsulation, can reduce moisture ingress and increase the operational lifespan of the cells. Development of policies and market strategies that promote the adoption of eco-friendly technologies like natural dye-based DSSCs can accelerate their commercialization. This could include incentives for research, development, and deployment, as well as standards for performance and durability.

If developed effectively, light-emitting plants could revolutionize outdoor lighting by providing illumination visible to the naked eye at night. These plants could also replace electric street lamps in regions without access to electricity, thereby reducing carbon dioxide emissions associated with conventional lighting methods. Additionally, they can offer aesthetic benefits as decorative elements, potentially enhancing urban and rural landscapes while contributing to environmental sustainability.

Let us not forget that plants represent a real treasure, because they can save our lives. Scientific research must spotlight the valorization of plants, phytowastes, and phytomolecules.

Bearing this in mind, we must learn to respect nature, to protect the “green gold”, by adopting “green” strategies in all areas, learn to recycle to protect the trees, and learn not to randomly throw away various things or polluting agents.

## Figures and Tables

**Table 1:** Advantages and disadvantages of plant decellularization methods.

Method	Advantages	Disadvantages	Ref.
Chemical-based	- High decellularization efficiency - Widely accepted as the gold standard - Effective at lysing cell membranes by targeting lipid–lipid and lipid–protein interactions	- Potential damage to ECM components - Requires careful optimization of concentration and exposure time - Some chemicals may be cytotoxic or difficult to remove completely from the tissue	[122]
Physical	- Minimal chemical exposure, reducing the risk of ECM damage - Can be used for tissues with complex structures - Less cytotoxicity compared to chemical methods	- Less efficient than chemical methods, may require longer processing times - Risk of mechanical damage to tissue structure - Limited effectiveness for tissues with dense matrices or extensive cross-linking	[123]
Enzymatic	- Specific enzymatic activity, targeting nucleic acids or proteins - Minimal damage to ECM components - Can be used for tissues with complex structures - Less cytotoxicity compared to chemical methods	- Enzymes may be expensive or difficult to source in large quantities - Optimization of enzyme concentration and activity required - Enzymatic reactions may be slow, necessitating longer processing times - Risk of incomplete removal of cellular debris	[122]

**Table 2:** A synopsis of select plant-derived scaffolds and their prospective utility in bone tissue engineering.

Plant Type	Treatment/Coating	Cells	Scaffold Properties	Outcome	Potential Application	Ref.
Onion (*Allium cepa*)	-	hBM-MSC	Low surface roughness, high and regular porosity	Enhanced osteogenic differentiation	Non-load bearing	[161]
Carrot (*Daucus carota*)	Poly-L-lysine	MC3T3	Heterogeneous structure, non-homogeneous pores distribution	Enhanced pre-osteoblasts adhesion proliferation and osteogenic differentiation	Bone filler, non-load bearing	[161]
Asian palmyra palm (*Borassus flabellifer*)	Organosilanes (APTES, OTS)	MG63	Fibrous interconnected structure	Improved osteoblast differentiation and mineralization	Bone filler, non-load bearing	[140]
*Alstroemeria* flower stem	Chitosan	MC3T3	Innate vasculature, high surface area	Improved mechanical behavior	Fluid transfer in BTE	[147]

## Data Availability

Not applicable.
